# Intermolecular Gene Conversion for the Equalization of Genome Copies in the Polyploid Haloarchaeon *Haloferax volcanii*: Identification of Important Proteins

**DOI:** 10.3390/genes15070861

**Published:** 2024-07-01

**Authors:** Hanna Özer, Daniel Wasser, Lara Sandner, Jörg Soppa

**Affiliations:** Biocentre, Institute for Molecular Biosciences, Goethe University, Max-von-Laue-Str. 9, D-60439 Frankfurt, Germany; hannaaylin.oezer@ukmuenster.de (H.Ö.); wasser@bio.uni-frankfurt.de (D.W.); larasandner@gmail.com (L.S.)

**Keywords:** archaea, *Haloferax volcanii*, polyploidy, gene conversion, homologous recombination, DNA repair, holiday junction, MutL, Hjc, NucS

## Abstract

The model haloarchaeon *Haloferax volcanii* is polyploid with about 20 copies of its major chromosome. Recently it has been described that highly efficient intermolecular gene conversion operates in *H. volcanii* to equalize the chromosomal copies. In the current study, 24 genes were selected that encode proteins with orthologs involved in gene conversion or homologous recombination in archaea, bacteria, or eukaryotes. Single gene deletion strains of 22 genes and a control gene were constructed in two parent strains for a gene conversion assay; only *radA* and *radB* were shown to be essential. Protoplast fusions were used to generate strains that were heterozygous for the gene *HVO_2528*, encoding an enzyme for carotinoid biosynthesis. It was revealed that a lack of six of the proteins did not influence the efficiency of gene conversion, while sixteen mutants had severe gene conversion defects. Notably, lack of paralogous proteins of gene families had very different effects, e.g., mutant Δrad25b had no phenotype, while mutants Δrad25a, Δrad25c, and Δrad25d were highly compromised. Generation of a quadruple *rad25* and a triple *sph* deletion strain also indicated that the paralogs have different functions, in contrast to *sph2* and *sph4*, which cannot be deleted simultaneously. There was no correlation between the severity of the phenotypes and the respective transcript levels under non-stressed conditions, indicating that gene expression has to be induced at the onset of gene conversion. Phylogenetic trees of the protein families Rad3/25, MutL/S, and Sph/SMC/Rad50 were generated to unravel the history of the paralogous proteins of *H. volcanii*. Taken together, unselected intermolecular gene conversion in *H. volcanii* involves at least 16 different proteins, the molecular roles of which can be studied in detail in future projects.

## 1. Introduction

Gene conversion is defined as the non-reciprocal flow of information between two homologous but not identical DNA sequences. Gene conversion occurs in all three domains of life, archaea, bacteria, and eukaryotes. By far the most studies have been performed with eukaryotes, which have led to several thousand publications. In eukaryotes, gene conversion is involved, e.g., in meiosis and the development of the immune system. However, gene conversion in eukaryotes is not part of this study, and reviews about various aspects are available for those interested in this topic [1,2,3,4,5,6,7,8].

Gene conversion in bacteria is involved in several biological processes. One such process is antigenic variation, which enables pathogens to escape the immune system of the host [9]. To this end, the genome of the pathogen contains one expression site for a surface protein and several to many silent paralogous genes lacking a promoter. With low frequencies, the gene at the expression site is converted by one of the silent genes, leading to a change in the surface structure of the pathogen, which makes the immune response obsolete. The number of silent copies can vary widely even within one genus, e.g., there are about 6 silent copies in *Anaplasma marginale* and more than 100 silent copies in *A. phagocytophilum* [10]. The eukaryotic pathogen *Trypanosoma brucei* contains even more than 1500 silent copies of the variant surface glycoprotein [11]. In antigenic variation, gene conversion results in generating diversity within the population of pathogens. The number of possible variants is even much higher than the number of silent sites because the gene conversion tracts are very short and therefore only parts of the silent copies are involved in gene conversion, leading to novel hybrid genes at the expression site. At least in bacteria, gene conversion during antigenic variation occurs at different sites of the same chromosome and is thus an intramolecular event.

Another example for intramolecular gene conversion is the concerted evolution of gene families, which also occurs in species of various phylogenetic groups [9,12,13]. In stark contrast to antigenic variation, gene conversion in concerted evolution leads to the equalization of several copies of a gene that are present in one genome. One example are the genes for the ribosomal RNAs (rRNAs). Most bacteria contain more than one rRNA operon [12]. For example, *Escherichia coli* contains seven rRNA operons, and in other bacterial species the number can be as high as fifteen. Concerted evolution has also been observed in families of protein-coding genes, e.g., the *tuf* genes in *Salmonella* or the *nif* genes in *Rhizobium* [14,15]. Also, in concerted evolution, the gene conversion frequencies are very low, so that selection steps have to be involved for the identification and characterization of gene conversion events, and similar to antigenic variation, the gene conversion tract lengths in concerted evolution are rather short, so that only fractions of genes are converted in one event. On the one hand, concerted evolution can correct mutations that have occurred in a single of several gene copies and preserve the wildtype sequence, while on the other hand, it can also spread advantageous mutations from the gene copy in which they have occurred to the other copies of the gene family. 

Another process that results in equalization of gene copies is intermolecular gene conversion between different copies of the chromosome in polyploid prokaryotes. Until now, this process has only been studied in halophilic archaea [16,17,18], in methanogenic archaea [19], in chloroplasts [20,21,22,23], and in mitochondria [24,25,26]. Intermolecular gene conversion in polyploid bacteria has not been studied yet. However, it can be expected to also operate in bacteria and to result in the equalization of genome copies, because (1) genome sequencing projects yield unambiguous sequences and do not point to heterozygosity, (2) heterozygous cells have been obtained with several bacterial species, but laboratory selection was needed for their generation [27,28,29,30], and (3) homozygous mutants can easily be generated in polyploid archaea and bacteria, although initially only one of many copies is mutagenized [31,32]. However, one exception has been reported, the giant bacterium *Achromatium oxaliferum* was shown to be naturally heterozygous [33].

In contrast to antigenic variation and concerted evolution, intermolecular gene conversion in polyploid archaea has a high efficiency, which enables its direct characterization without a selection step that enriches for a very seldom event. Recently, an experimental approach for studying unselected intermolecular gene conversion between different genome copies has been reported for *H. volcanii* [18]. To this end, two strains were generated that contained two different versions of a gene encoding an enzyme involved in carotinoid biosynthesis, i.e., an active version leading to red cells and an inactive version leading to white cells. The two strains were combined via protoplast fusion, resulting in heterozygous cells containing both types of genomes. The efficiency of gene conversion can be analyzed via the color of the resulting recombinants (cells that became homozygous for the inactive copy are white) or via PCR analyses. The analyses not only confirmed the high efficiency of gene conversion but also revealed that the conversion tracts are much longer than in antigenic variation or concerted evolution and can in fact span several thousand base pairs [18]. In addition, it was found that gene conversion was triggered by very small differences between the genome copies and that even sequences from very different species become converted.

In the present study, we applied this approach to identify proteins that are involved in and important for intermolecular gene conversion in *H. volcanii*. More than 20 genes were selected that encoded proteins that might be involved in homologous recombination or different DNA repair pathways. Single gene deletion mutants of all genes (with the exception of two essential genes) were generated, and the effect on the efficiency of gene conversion was quantified. In addition, one triple mutant and one quadruple mutant of paralogous genes were also generated and tested. The results revealed that several proteins did not affect the efficiency of gene conversion, while the majority had a very profound effect. To unravel the history of the paralogous *H. volcanii* members of three protein families, phylogenetic trees were generated with proteins from archaea, bacteria, and eukaryotes.

## 2. Materials and Methods

### 2.1. Strains, Media, and Growth Conditions

All strains used in this study are derivatives of the *H. volcanii* wildtype DS2 [34]. All strains contain a deletion of the *pyrE2* (*HVO*_0333) encoding an enzyme for uracil biosynthesis, which is necessary for the efficient construction of further mutants [35]. One of the two parent strains (H53) used for protoplast fusion (see below) contains a further deletion in *trpA* (*HVO_0789*), making it auxotrophic for tryptophan [31]. The other parent strain (So0994) contains a deletion in *thyA*, making it auxotrophic for thymidine, and *crtD* (*HVO_2528*), leading to white colonies. Further mutants were generated in the framework of this project, as described below.

The *H. volcanii* strains were grown in complex medium or in synthetic medium with 0.5% (*w*/*v*) glucose as sole carbon and energy source [36]. If necessary, the medium was supplemented with 50 µg/mL uracil, 50 µg/mL tryptophan, or/and 20 µg/mL thymidine to allow the growth of auxotrophic strains. The cultures were grown at 42 °C with good aeration (250 rpm). Solid media contained 1.4% (*w*/*v*) agar.

The *E. coli* strain XL1-blue MRF’ (Agilent Technologies, Waldbronn, Germany) was used for cloning. It was grown in SOB complex medium [37].

### 2.2. Generation of In-Frame Deletion Mutants

The in-frame deletion mutants were generated using the so-called Pop-In-Pop-Out method as described previously [31,38]. In short, for each gene two PCR fragments were generated that contained, respectively, the upstream region and a short 5′-part of the gene, and a short 3′-part of the gene and the downstream region. All primers are listed in Supplementary Table S1. The two PCR fragments were fused and cloned into the vector pMH101 via restriction selection cloning [39]. The resulting plasmids were verified by sequencing and used to transform the two parent strains H53 and So0994. Cultivation in medium lacking uracil selected for clones that had integrated the plasmids at the respective genomic sites (Pop-In). Subsequent cultivation of the Pop-In clones in medium with 5-FOA (5-fluororotic acid) and uracil selected for clones that had lost the integrated vector based on a second recombination event (Pop-Out). Colony PCR was used to identify clones that contained the deletion version in the genome. Because *H. volcanii* is polyploid, clones can be heterozygous in spite of the high efficiency of gene conversion. Therefore, genomic DNA was isolated and analyzed via Southern blotting and with a PCR with 40 cycles. Table 1 summarizes the 23 genes that were deleted in both parent strains. 

### 2.3. Growth Analyses

*H. volcanii* can be grown in microtiter plates, which enables the characterization of many strains in parallel [36]. Growth of all 46 strains was monitored in complex medium as well as in synthetic medium with 0.5% (*w*/*v*) glucose as the sole source of carbon and energy. In short, precultures were grown to the mid-exponential growth phase and used to inoculate test cultures with an OD_600_ of 0.05. The microtiter plates were incubated at 42 °C on a Heidolph Titramax 1000 rotary shaker with 1100 rpm. The OD_600_ was determined at the indicated time points using a Spectramax 340 photometer (Molecular Devices, Ismaning, Germany). Three biological replicates were performed, and average values and their standard deviations were calculated. 

### 2.4. Analyses of Gene Conversion Efficiencies

To test the possible influence of the 22 selected proteins and the control protein DHFR (Table 1) on the efficiency of intermolecular gene conversion, pairs of cultures were grown to mid-exponential growth phase that contained the deletion of a specific gene in the two parent strains H53 and So0994. A total of 8 × 10^8^ cells of both strains were fused by protoplast fusion as described earlier [18,40,41]. Fused cells were selected in synthetic medium lacking tryptophan and thymidine, which does not allow growth of the parent strains. This generates cells that contain about 20 copies of each of the two genomes, which contained a wildtype copy of the gene *HVO_2528* (carotinoid biosynthesis proficient) and a deletion version of the gene (carotinoid biosynthesis deficient). Directly after protoplast fusion, many clones are still heterozygous, and plating results in a high fraction of sectored colonies. To allow completion of gene conversion, the mixture of fused cells was inoculated in 30 mL synthetic medium and incubated for 24 h. Then, serial tenfold dilutions were generated and spread on agar plates with synthetic medium. After five days of incubation at 42 °C, colonies had been formed. The plates were moved to room temperature and incubated for a further 5 days, which facilitates the differentiation between white colonies, red colonies, and sectored colonies. The three types of colonies were counted separately using plates with a suitable dilution (50–500 colonies per plate), and the copy number of the undiluted culture was calculated. At least four biological replicates of all protoplast fusion were performed, and average values and their standard deviations were calculated. The numbers were normalized to the copy numbers of control protoplast fusions with the two parent strains. The significance of observed values between the test strains and the parent strains was calculated using an unpaired two-tailed *t*-test. 

### 2.5. Bioinformatic Analyses

The genome database Halolex [42] was used to retrieve sequences from the genome of *H. volcanii* and to address the newest genome annotation. The clone manager Professional Suite version 8 (Sci Ed Software, Westminster, CO, USA) was used for the experimental design. The program MEGA X with the algorithm MUSCLE was used to generate multiple sequence alignments of various orthologues and paralogues of the studied proteins. The phylogenetic trees were constructed on the basis of multiple sequence alignments. The program MEGA X [43] was used for tree construction, and in each case the maximum likelihood, maximum parsimony, and neighbor-joining approaches were used. A total of 1000 bootstrap repetitions were performed, and the results (%) were added to selected nodes. In addition, individual neighbor-joining trees were generated, which allowed us to include the actual branch lengths.

The results from previous RNA and dRNA sequencing studies [44,45] were analyzed to gain insights into the expression of selected genes. The Integrated Genome Browser [46] was used to visualize the data.

## 3. Results

### 3.1. Experimental Design for the Analysis of Unselected Gene Conversion

**Figure 1:**
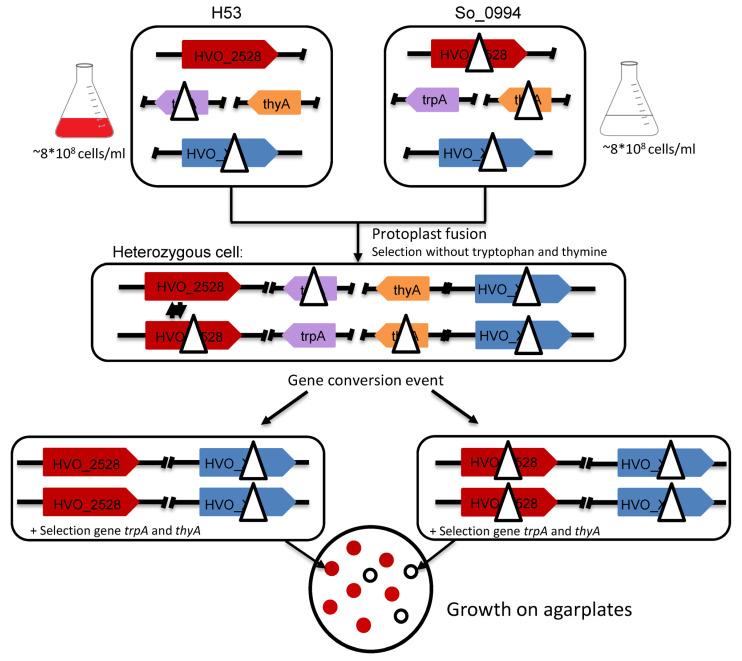
Schematic overview of the experimental design of gene conversion experiments with *H. volcanii*. Two parent strains with nearly identical genomes are generated, the only difference is that H53 contains a deletion in the *trpA* gene and So_0994 carries deletions in the genes *thyA* and *HVO_2528*, as indicated. To test the possible importance of selected proteins on the efficiency of gene conversion, the respective genes (indicated in blue) were deleted in both parent strains. The two parent strains were fused by protoplast fusion, and fused heterozygous cells (middle) were selected by growth in the absence of tryptophan and thymidine. After gene conversion, two different homozygous cells (concerning *HVO_2528*) are possible, which are indicated in the lower part of the figure. The genes are color-coded to enable an easy overview.

Recently, we have developed an experimental approach to quantify the efficiency of unselected intermolecular gene conversion in *H. volcanii* [18]. An extended version was used to analyze the importance of more than 20 selected proteins on gene conversion. A schematic overview of the approach is given in Figure 1. In short, two parent strains were generated that have an intact or an inactivated version of the gene *HVO_2528*, respectively. The gene encodes an enzyme involved in carotinoid biosynthesis and the strains are thus red and white, respectively. In addition, the strains contain a deletion either in *trpA* (essential for tryptophan biosynthesis) or *thyA* (essential for thymidine biosynthesis). In the absence of tryptophan and thymidine, neither of the two parent strains can grow. However, after protoplast fusion of the two parent strains, heterozygous fusion cells contain a wildtype copy of both *trpA* and *thyA*, are prototrophic for tryptophan and thymidine, and can be selected for in the absence of both substances. Unselected gene conversion can occur at the *HVO_2528* locus either in the direction of the wildtype version or in the direction of the deletion version. If the deletion version becomes homozygous, the cells become white. Therefore, the fraction of white colonies can be taken as a quantitative value for the efficiency of gene conversion in the direction of the deletion version. This allows the very fast and easy analysis of gene conversion efficiencies in one direction in thousands of colonies. In contrast, red colonies can either be heterozygous (as directly after protoplast fusion) or homozygous for the wildtype version after gene conversion in this direction. To discriminate between these two possibilities, PCR analysis has to be applied. As colony PCR is established for *H. volcanii*, hundreds (but not thousands) or clones can easily be analyzed.

For the analysis of the importance of selected proteins for intermolecular gene conversion, the cognate genes have to be deleted in both parent strains. After protoplast fusion, the fusion cells are heterozygous for *HVO_2528* but homozygous for the deletion version of the gene under investigation, and the effect of the absence of the encoded protein can be quantified. Initially, it was expected that the fraction of white colonies could be taken as readout. However, during the course of the project, it turned out that the number of surviving cells was more informative (see below). 

### 3.2. Overview of Generated and Analyzed In-Frame Deletion Mutants

A literature search was performed to identify proteins that have been shown to be involved in “gene conversion” or “homologous recombination” in archaea, bacteria, or eukaryotes. If a clear homolog was encoded in the genome of *H. volcanii*, the respective gene was considered as a candidate for the analysis. The gene *dhfr*, encoding dihydrofolate reductase, was added as a negative control because it was assumed that this metabolic enzyme should not play a role in the molecular mechanism of gene conversion. Table 1 gives an overview of the selected genes and the annotated functions of the encoded proteins. 

**Figure 2:**
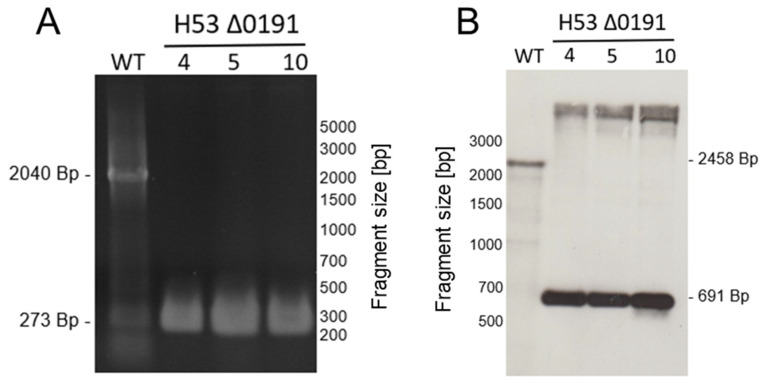
Verification of homozygosity of newly generated deletion mutants. The deletion mutants were compared with the wildtype using two different approaches, i.e., multicycle PCR and Southern blotting. (**A**) Results of a multicycle PCR for the wildtype and three clones of mutant Δ0191. (**B**) Results of a Southern blot for the wildtype and three clones of mutant Δ0191. Mutant Δ0191 in the parent strain H53 is shown to exemplify the analyses. The two analyses were performed for all 46 mutants.

Single gene deletion mutants were generated for the 23 genes in both parent strains H53 and So_0994, resulting in a set of 46 strains. Only the genes *radA* and *radB* could not be deleted and were thus regarded as essential. The so-called Pop-In-Pop-Out method was applied for mutant construction [31,39,47]. Supplementary Table S1 lists the primers that were used for mutant generation. *H. volcanii* is polyploid with about 20 copies of its major chromosome [48]; therefore, deletion strains sometimes retain one or a few copies of the wildtype allele. To exclude this possibility, all deletion mutants were checked with both PCR analysis with 40 cycles and with Southern blot analysis. The analysis of deletion mutant ΔHVO_0191 is shown in Figure 2A (PCR analysis) and Figure 2B (Southern blot analysis) to exemplify the results. Homologous deletions could be verified for all 46 strains.

### 3.3. Growth Analysis of Deletion Mutants

**Figure 3:**
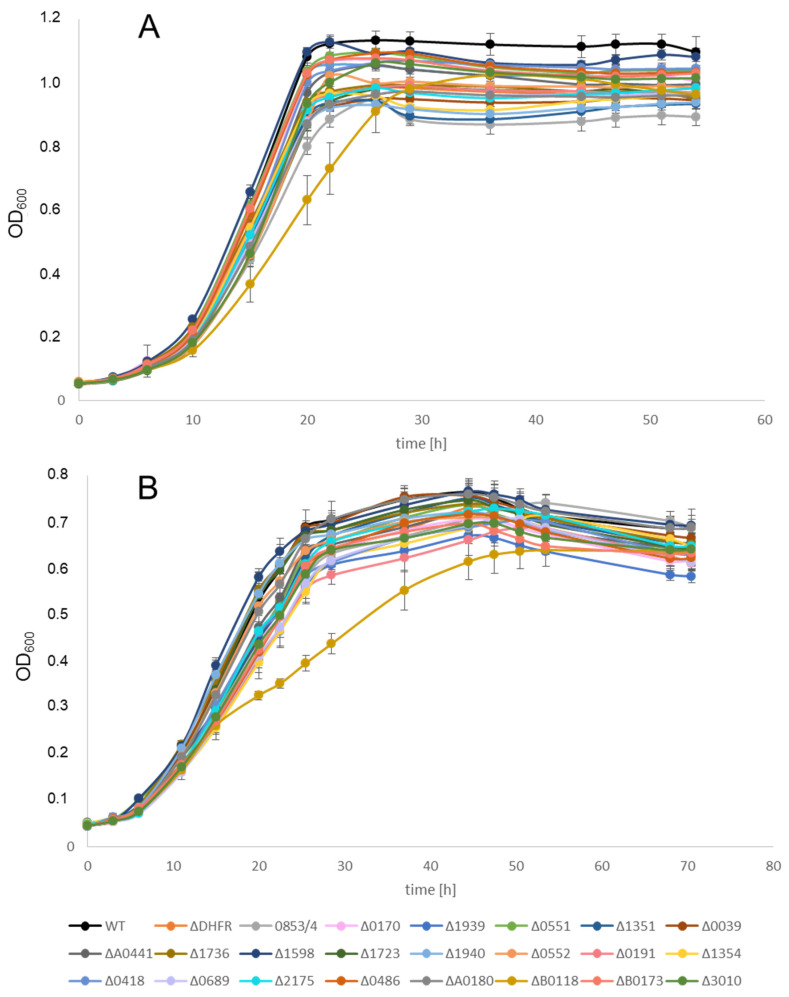
Growth curves of the wildtype and 23 deletion mutants (in the parent strain H53). The growth was performed in microtiter plates. Three biological replicates were performed. Average values and their standard deviation are shown. (**A**) Growth in complex medium. (**B**) Growth in synthetic medium with glucose as carbon and energy source.

Growth of all deletion strains was compared with that of the wildtype. To this end, the strains were grown in microtiter plates as described [36]. Three biological replicates were performed. Figure 3A shows the results of the growth experiments for the 23 deletion mutants in the parent strain H53 in complex medium. With one exception, all strains grew indistinguishably. Only deletion strain Δ*HVO_B0118*, lacking the gene for the protein Sph2, had a slightly reduced growth rate, but it reached the same growth yield as all other strains. The same result was obtained with the 23 deletion strains in the parent strain So_0994. Figure 3B shows the growth of the 23 deletion mutants and the wildtype in synthetic medium with glucose as the carbon and energy source. This was tested because the selection after protoplast fusion has to be performed in synthetic medium. Again, only strain Δ*HVO_B0118* had a slight growth defect, while all other strains grew indistinguishably. The same result was obtained with the 23 deletion strains in the parent strain So_0994. These results revealed that under optimal growth conditions, none of the 22 DNA repair and recombination proteins is so important that its absence results in a severe growth defect. Of course, the importance of several of these proteins might be much higher after the application of mutagenic conditions or stress, which, however, were not part of the current project. Therefore, all mutants were suited to analyzing the effect of the lack of the proteins on the efficiency of gene conversion.

### 3.4. Gene Conversion Efficiencies of Wildtype and Mutants

**Figure 4:**
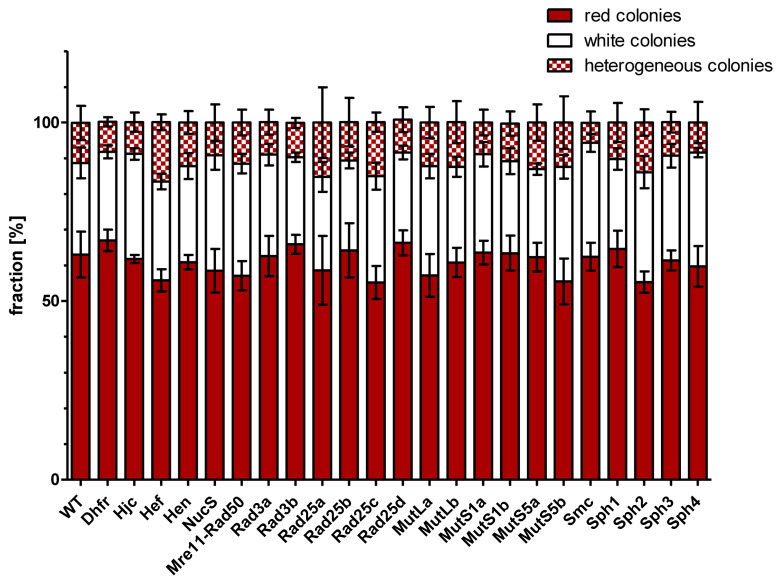
The fractions of red, white, and sectored colonies after gene conversion experiments are shown for the parent strains (wt) and 23 deletion mutants. Average values of four biological replicates and their standard deviation are shown.

Gene conversion experiments were performed with the two parent strains and the 23 deletion mutants of each of the two parent strains. The generation of—very sensitive—protoplasts, their fusion in the presence of PEG, and the selection of fusion cells might have a higher variance than other methods that do not compromise the integrity of the S-layer; therefore, four biological replicates were performed for each gene conversion experiment. It was expected that lack of a protein with an important function for gene conversion would lead to a lower gene conversion efficiency, which would result in a lower fraction of white colonies (with complete conversion of *HVO_2528* to the deletion variant). Surprisingly, this was not observed. Figure 4 summarizes the fractions of red, white, and sectored colonies after all 24 gene conversion experiments. There was no significant difference between the 2 controls (parent strains, parent strains lacking the metabolic enzyme DHFR) and the 22 gene conversion experiments with strains lacking a protein annotated to be involved in homologous recombination or DNA repair. It is highly unlikely that none of the 22 proteins are involved in gene conversion; therefore, the fraction of white colonies was obviously not informative about the efficiency of gene conversion, in contrast to the expectation.

However, we noticed that the number of clones that survived the gene conversion experiments was highly variable and that the reduced survival rates were clone-specific and highly reproducible. The possibility can be excluded that the allele of the carotenoid biosynthesis gene *HVO_2528* was related to the differences in survival because the two parent strains with the native and the inactivated allele grew equally well and because carotenoids do not have a vital function during non-stressed growth in the dark. The most plausible explanation is that our initial expectation was wrong that the absence of an important protein should result in a higher fraction of cells that do not experience gene conversion and remain heterozygous. It seems that all heterozygous fusion cells had initiated gene conversion. Gene conversion includes the interconnection between two genome molecules that act as donor and acceptor. If gene conversion cannot be terminated because an important protein is missing, these intertwined molecules might interfere with replication, genome segregation, and cell division. This might lead to cell death or at least prevent the outgrowth of clones that have initiated but failed to terminate gene conversion, resulting in reduced apparent survival rates in the gene conversion assay.

**Figure 5:**
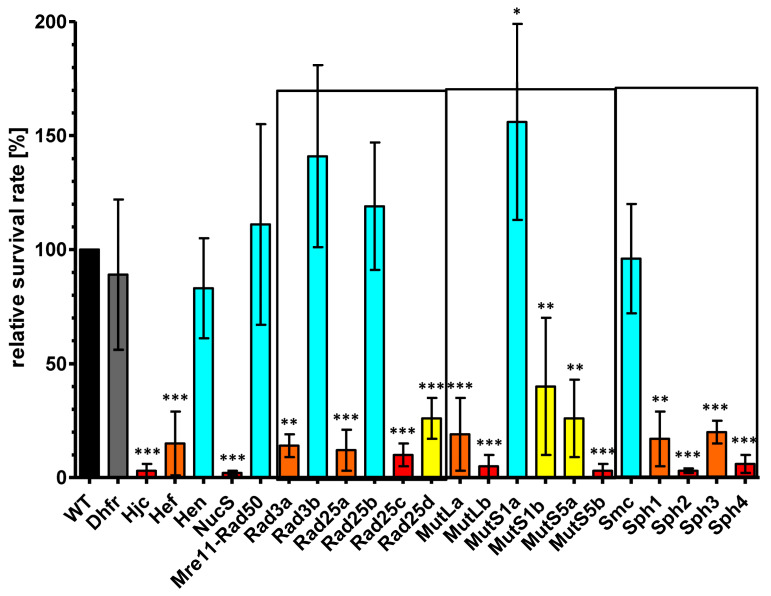
Normalized survival rates after gene conversion experiments are shown for the parent strains (normalized to 100%) and 23 deletion mutants. Families of paralogous proteins are boxed. The significances of the observed differences were calculated using an unpaired two-tailed *t*-test, with the following *p*-values: * < 0.05, ** < 0.01, *** < 0.001.

Therefore, the numbers of surviving cells were taken as an alternative readout for the efficiency of unselected intermolecular gene conversion. Supplementary Table S2 lists the average values and their standard deviations of the four biological replicates (more than 20 replicates for the wildtype), and Figure 5 visualizes the results after normalization to the control gene conversion experiment with the two parent strains. The survival rate of the control lacking the DHFR was very similar to that of the two parent strains. The same was true for six of the test strains, indicating that the proteins lacking in these strains are not involved in or at least not important for gene conversion. In stark contrast, seven of the test strains had severe deficits, with survival rates of 10% or less compared to that of the parent strains (dark red in Figure 5). In all cases, the differences compared to the control were highly significant (*p* < 0.001). In an additional nine cases, the survival rates were between 10% and 40% compared to the control (light red and yellow in Figure 5). Depending on the variance, the differences were significant (*p* < 0.01) or highly significant (*p* < 0.001), as indicated. Noteworthy is the fact that, in several cases, the results were very different for paralogous proteins of the same family, e.g., the lack of one of the four paralogs of Rad25 did not result in reduced survival (Rad25b) or resulted in normalized survival rates of 26%, 12%, and 10%, respectively. In addition, the lack of SMC did not influence survival, while the lack of the four SMC-like proteins (Sph1–4) resulted in drastic reduction of the survival rates down to about 20% (Sph1 and Sph3) or to about 5% (Sph2 and Sph4). Taken together, the gene conversion assay resulted in drastically different survival rates, and a lack of 16 of the 22 selected proteins had a profound effect. If the harsh treatment of the cells in the assay is taken into account, the reproducibility of the four biological replicates was quite high in the majority of cases, with a few exceptions. 

### 3.5. Analysis of Gene Expression

Recently, we performed a dRNA-Seq analysis to obtain an overview of transcription start sites and a mixed RNA-Seq analysis to obtain an overview of the transcriptome of *H. volcanii* [44,45]. The mixed RNA-Seq results represent four conditions, i.e., exponential growth in complex and synthetic medium at the optimal salt concentration, exponential growth at low salt, and stationary phase in complex medium. The results for all genes in this study were analyzed to reveal whether the genes are expressed under non-stressed conditions and whether any correlation exists between the severity of the effects and the expression levels. Screenshots for the first four proteins are shown in Supplementary Figure S1. In these and the following Figures, the annotated genes are shown in blue, the dRNA-Seq results in green, and the RNA-Seq results in red. The genes *hjc* and *nucS* were not expressed under the analyzed conditions, while *hef* and *hen* were highly expressed. The results for the *rad* genes are shown in Supplementary Figure S2. Of the four *rad25* paralogs, only *rad25b* is highly expressed, while the deletion mutant Δ*rad25b* had no gene conversion defect. The expression levels of the other three paralogs are very low, while all three respective deletion mutants had severe survival defects. Similarly, the transcript level of *rad3b* under the tested conditions was high, while the deletion mutant had no deficit, and the opposite was true for *rad3a*. The results for the *mut* genes are shown in Supplementary Figure S3. The expression level of all six genes was very low, while the survival rates of the respective deletion mutants varies from 3% to 156%. The results for the *sph* genes (+ *smc + rad50*) are shown in Supplementary Figure S4. Only the genes *smc* and *sph2* were highly expressed, while the other four genes had a rather low expression level. Taken together, there was no correlation between the transcript levels and the severity of the gene conversion defect in the respective deletion mutants. Thus, expression of the 22 genes under the four conditions tested in mixed RNA-Seq was not indicative for the importance of the encoded proteins for gene conversion. It might be assumed that the expression of the genes important for gene conversion has to be induced when the process is initiated.

### 3.6. Bioinformatic Analyses of Paralogous Protein Families

**Figure 6:**
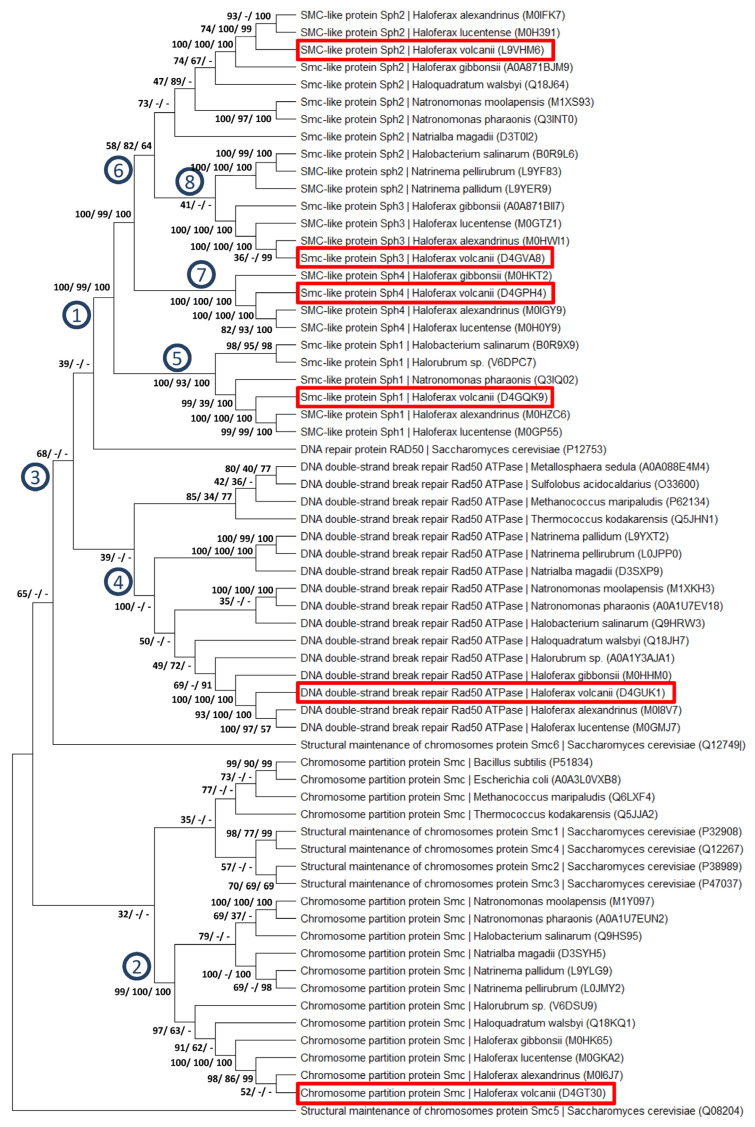
Consensus phylogenetic tree of the Sph/SMC/Rad50 protein family. Selected proteins from archaea, bacteria, and eukaryotes were used to construct a multiple sequence alignment. The alignment was used to generate the phylogenetic tree using the neighbor-joining option of the program MEGA X [43]. A total of 1000 bootstrap replications were performed and the bootstrap values (%) are shown at the respective nodes. Trees with 1000 bootstrap replications were also generated using the maximum parsimony and the maximum likelihood algorithms, and the bootstrap values are also included in the figure (in the order maximum likelihood/maximum parsimony/neighbor-joining) The numbered nodes are discussed in the text. The paralogs from *H. volcanii* are highlighted by red boxes.

Various genes included in this study encoded paralogous members of protein families. In three cases, multiple sequence alignments were generated and used to construct phylogenetic trees. Figure 6 shows a tree of the Sph protein family together with SMC and Rad50 proteins. It is a consensus tree obtained after 1000 bootstrap replications using the maximum likelihood algorithm. At selected nodes, the bootstrap values (%) are indicated that were obtained using the maximum likelihood, maximum parsimony, and neighbor-joining algorithms, respectively. An individual neighbor-joining tree is shown in Supplementary Figure S5, which includes information about the branch length but does not include information about the robustness of the tree. 

The trees contain 64 proteins, mostly from halophilic Archaea, but also from other Archaea, Bacteria, and Eukaryotes. “SMC-like” (Sph) proteins occur only in halophilic archaea. All Sph proteins originated from on common ancestor (node 1 in Figure 6). All haloarchaeal SMC proteins also form a coherent group (node 2) but are rather far from the Sph proteins. The support for the monophyly of all archaeal, bacterial, and eukaryotic SMC proteins is somewhat lower (node 3), probably due to eukaryotic proteins SMC5 and SMC6, which are distant from all other SMC proteins. Similarly, all haloarchaeal Rad50 proteins form one well-supported group (node 4). The monophyly of all Rad50 proteins is not well supported, because the distinct position of the eukaryotic ortholog. Within the group of Sph proteins, two well-supported subgroups were found, on the one hand Sph1 (node 5), and on the other hand Sph2-4 (node 6). Within the latter group, the support for the monophyly of Sph3 and Sph4 is high (nodes 7 and 8), while the Sph2 do not form a monophyletic group. 

A Rad25 maximum likelihood consensus tree including the bootstrap values of all three approaches is shown in Supplementary Figure S6. An individual neighbor-joining tree is shown in Supplementary Figure S9. The numbers of Rad25 paralogs vary widely, e.g., *H. volcanii* contains four paralogs, while *Haloferax mediterranei* from the same genus contains only two paralogs. Designation of the paralogs during genome annotation was typically not based on phylogenetic analysis, but two paralogs were named a and b and four paralogs were named a to d. Therefore, paralog designations are not informative about phylogenetic relationships. The tree indicates that the last common ancestor of all haloarchaea contained two paralogs (node 1 and 2 Supplementary Figure S6). In the lower part of the tree, which contains the *H. volcanii* paralog Rad25d, no further gene duplications occurred. However, during further evolution of halophilic archaea another gene duplication occurred, which can be observed in the upper part of the tree (node 3 and 4). In contrast to most other species, *H. volcanii* experienced an additional gene duplication (node 5), which generated Rad25a and Rad25b. 

A maximum likelihood consensus tree of selected Mut proteins is shown in Supplementary Figure S8. An individual neighbor-joining tree is shown in Supplementary Figure S9. The upper part contains all prokaryotic MutL sequences together with their eukaryotic homologs that are named MLH (node 1), while the lower part contains all MutS sequences together with their eukaryotic homologs named MSH (node 2). In the MutL branch, a gene duplication occurred and resulted in the two paralogs, MutLa and MutLb, in genera like *Haloferax* and *Haloquadratum*, which did not occur in genera like *Natrialba*, *Natrinema* or *Natronomonas*. In the MutS branch, a gene duplication in archaea led to the split into the MutS5 group (node 3) and the MutS1 group including the eukaryotic MSHs (node 4). The latter group divided into the archaeal MutS1 group (node 5) and the eukaryotic MSH group (node 6). In each of the two archaeal branches, a further gene duplication event led to the generation of two further paralogs (nodes 7 + 8 and nodes 9 + 10). This evolutionary scenario can explain the occurrence of six Mut paralogs in *H. volcanii*. 

Taken together, in all three protein families, like in other protein families present in *H. volcanii*, the present paralogs arose through gene duplications that occurred at very different phylogenetic levels, some of which are confined to single genera or even species. Haloarchaea and especially *H. volcanii* are characterized by the presence of a higher number of paralogs in many protein families than in other evolutionary lineages.

### 3.7. Generation and Characterization of Mutants with Multiple Deletions

*H. volcanii* has four paralogs of the Rad25 and Sph protein families; therefore, we decided to generate mutants with multiple deletions. This approach aimed to elucidate whether the paralogs have redundant functions and can functionally replace one another or whether all paralogs have evolved to fulfill different functions. In the former case, it can be expected that the survival rates would be much lower in multiple than in single mutants, while in the latter case, the survival rate of a multiple mutant should be similar to the lowest survival rate of any of the single mutants.

In the *rad25* family, it was indeed possible to generate quadruple mutants combining the four deletions in *rad25a-d* in both parent strains. The quadruple mutants did not exhibit a growth defect in complex or synthetic medium. A gene conversion experiment was performed with four biological replicates, and the survival rate was 14%. This survival rate was very similar to the survival rates of Δ*rad25a* and Δ*rad25c* (12% and 10%), the two paralogs with the lowest survival rates of the four single mutants. Therefore, it seems that the four Rad25 paralogs have evolved to fulfill separate functions and are not redundant to one another.

A quadruple deletion mutant of the *sph* genes could not be generated because it turned out that *sph2* and *sph4* cannot be deleted simultaneously. This indicates that Sph2 and Sph4 are at least partially redundant and fulfill an essential role in *H. volcanii* for growth and survival under optimal conditions. However, a triple Δ*sph1/3/4* could be generated, and the triple mutant did not exhibit any growth defect. In gene conversion experiments, the triple mutant had an average survival rate of 10% (± 7%), similar to the survival rates of the Δ*sph2* and Δ*sph4* single mutants with survival rates 3% and 6%. This indicates that Sph1 and Sph3 do not have overlapping functions with Sph2/4, at least in the molecular mechanism of intermolecular gene conversion. 

### 3.8. Further Characterization of the Sph-Deletion Mutants

Recently, it has been reported that Sph3 is involved in cell shape determination and that a Δ*sph3* mutant has a defect in swarming [49]. To unravel whether this might also be true for the other Sph proteins, the swarming abilities of the four single mutants and the triple mutant were compared to that of the wildtype. The results are shown in Supplementary Figure S10. In fact, the Δ*sph3* mutant (and the triple mutant) did not swarm at all for the first 48 h, in agreement with the recent report [49]. The swarming ability after 48 h differed for all four Δ*sph* mutants (Supplementary Figure S10). Notable, the lack of Sph4 led to a severe swarming deficit, while the lack of Sph2 resulted in an increase in swarming, compared to the wildtype. These results revealed that Sph2 and Sph4 have a redundant function in gene conversion (see above) but that they fulfill opposite functions during swarming. After 48 h, all mutants started to swarm with velocities not very different from that of the wildtype. 

It has also been reported that the swarming ability is correlated with the cell shape, i.e., that only rod-shaped cells are able to swarm [49]. In addition, it is known that wildtype cells are rod-shaped only in the very early exponential phase and transform to pleiomorphic cells during further growth [50]. Therefore, the cells shapes of the *sph*-deletion mutants were compared to that of the wildtype at three different times during the growth curve: at OD_600_ of 0.03 representing the very early exponential phase, at OD_600_ of 0.3 representing the mid-exponential phase, and at OD_600_ of 1.6 representing the stationary phase. The results are summarized in Supplementary Figure S11. In agreement with the recent report [49], we found that the *sph3*-deletion mutant does not form rods, even in the very early exponential phase. The opposite phenotype was observed for the *sph2*-deletion mutant, which formed predominantly rods even during the mid-exponential and stationary phases. These observations were in excellent agreement with the swarming deficit of Δ*sph3* and the over-swarmer phenotype of Δ*sph2* described above. The morphologies of the *sph1* and *sph4* were very similar to that of the wildtype. Similar to the results of the swarming assay, the cell shape analysis revealed that Δ*sph2* and Δ*sph4* had different phenotypes, in contrast to the gene conversion experiments, which revealed that the two genes are synthetically lethal. This can be taken as another indication that Sph2 and Sph4 have overlapping but not identical functions.

## 4. Discussion

The high efficiency of unselected intermolecular gene conversion enabled us to establish an experimental approach for the direct characterization of different aspects of gene conversion [18]. In stark contrast, experimental approaches for the characterization of intramolecular gene conversion in antigenic variation or concerted evolution of gene families required prior selection schemes because these processes occur only very rarely [9]. The current study aimed to unravel which of 22 proteins with DNA-related functions are involved in and important for gene conversion in *H. volcanii*. It had been expected that the absence of proteins with functional relevance would inhibit or slow down gene conversion and that this would lead to a higher fraction of heterozygous cells. Because completed gene conversion in the direction of the mutated copy of the reporter gene *HVO_2528* results in a loss of carotenoid biosynthesis, this should be directly visible based on a reduced fraction of white colonies. Unexpectedly, the fraction of white colonies was very similar in all mutants and the wildtype. PCR analyses of red colonies revealed that more than 98% of them were homozygous for the wildtype copy of *HVO_2528* and thus had experienced completed gene conversion (unpublished data). Therefore, the initially anticipated higher fraction of heterozygous cells that had not experienced gene conversion was not observed. This indicates that nearly all heterozygous cells obtained after protoplast fusion had initiated gene conversion. However, when an important protein was missing, the progression of gene conversion stalled and gene conversion intermediates remained, e.g., unresolved Holliday junctions. Such intermediates are probably incompatible with replication, genome segregation, and cell division. When such intermediates cannot be resolved at all, no apparent survivors of the gene conversion assay can be expected. In this model, the apparent survival rates are correlated to the remaining efficiency to resolve gene conversion intermediates and complete gene conversion in spite of the lack of an important protein. The higher the importance of the protein for the molecular mechanism of gene conversion in *H. volcanii*, the lower the normalized apparent survival rate should be.

In fact, it was found that lack of only 6 of the 22 proteins did not change the survival rate, while the lack of 16 of the 22 proteins led to a considerably reduced survival, which was less than 10% in 6 of the cases. This represents **the first comprehensive study addressing the importance of proteins for gene conversion in archaea**. A systematic generation of mutants has also been used for the identification of important proteins for antigenic variation in *Borrelia burgdorfei* (17 genes, [51]) and *Neisseria meningitides* (5 genes, [52]) or various steps in meiotic DNA processing in *Saccharomyces cerevisiae*, including gene conversion (81 genes, [53]).

Notably, in the current study, the ***radA*** gene could not be deleted. This result confirmed the results of earlier attempts of our group to delete the *radA* gene, which exclusively resulted in wild-type clones (in each case, more than 100 Pop_Out clones were screened; unpublished data). Therefore, in our hands, *radA* is an essential gene for *H. volcanii.* RadA is orthologous to the bacterial RecA protein and the eukaryotic Rad51 protein. Orthologs are present ubiquitously in all species of all three domains of life, and they are involved in the first step of homologous recombination, i.e., the search for homologous sequences in two DNA molecules [54,55]. Therefore, it is tempting to speculate that RadA should also be involved in early steps of gene conversion in *H. volcanii*, i.e., the search for homologous sites on different genome copies with sequence differences. However, despite the expectation that RadA or the bacterial ortholog RecA should be involved in gene conversion, this is not always the case. For example, antigenic variation in *B. burgdorfei* was found to be independent of RecA [56,57]. In contrast, RecA is essential for antigenic variation in *N. meningitides* [52]. Therefore, the necessity to clarify whether RadA is involved in gene conversion in *H. volcanii* persists, but alternative experimental approaches have to be used, e.g., the characterization of protein–protein interaction networks of important gene conversion proteins.

While the role of RadA remains unclear, the survival rates of six deletion mutants indicated that the encoded proteins are not involved in gene conversion or that their functions can be redundantly performed also be other proteins. The latter explanation might well hold true for three genes that are members of paralogous gene families, and these cases (*rad3b*, *rad25b*, *mutS1a*) are discussed below. However, this explanation is much less likely for the three single genes *hen*, *mre11-rad50*, and *smc* (discussed below). **Hen is a homing endonuclease**, and it has been shown that the Hen of *H. volcanii* is active and can insert an intein-encoding sequence into the chromosome by gene conversion [58]. While this results in the equalization of a intein-containing donor genome and a prior-to-intein-homing intein-lacking acceptor genome, this is a very specific activity, and it might not be surprising that Hen is not involved in general gene conversion. 

However, it was unexpected that the **Mre11-Rad50 protein complex** is not important for gene conversion in *H. volcanii*. The homologous eukaryotic Mre11-Rad50 complex is important for various processes involving gene conversion, e.g., one pathway (of several) of double strand break repair [59], immunoglobulin gene diversification [60,61], and recombination during meiosis [62]. Contrasting the roles in eukaryotes, the Mre11-Rad50 complex of *H. volcanii* has been shown to inhibit the repair of double strand breaks by homologous recombination, favoring repair by microhomology-mediated end-joining [55]. In addition, the complex is involved in nucleoid compaction after DNA damage, underscoring its involvement in DNA repair [63]. Our finding that Mre11-Rad50 is not involved in gene conversion indicates that this process is independent from double-strand break-induced repair pathways. 

Importantly, the current study revealed that 16 of the gene deletion mutants had severe defects in gene conversion. Three genes were single genes, i.e., *hjc, hef,* and *nucS*. **Hjc is a holiday junction resolvase** that is highly conserved in archaea. It specifically binds to four-way-junction DNA structures and induces endonucleolytic cleavages to initiate junction resolution. The crystal structures of Hjc orthologs from several different archaea have been solved, and models for the DNA binding have been generated [64,65,66,67]. In addition, biochemical and genetic studies with enzymes from different archaeal species have been performed [68,69,70,71,72]. This biological role fits well to the nearly complete loss of gene conversion in the *hjc*-deletion mutant (Figure 5).

Another protein that acts on branched DNA structures is **Hef (helicase-associated endonuclease for fork-structured DNA).** Hef is conserved in archaea and has been shown to be involved in the repair of stalled replication forks in various archaeal species [73,74,75,76]. The *H. volcanii* Hef has been characterized with genetic and biochemical approaches, and a review by Lestini et al. gives an overview of the results [77]. Eukaryotes contain a homolog to the archaeal Hef protein, and the human protein is called FANCM, because it is one protein of a protein complex that is related to Fanconi anemia [78,79]. Fanconi anemia is a recessive genetic disease that is characterized by genomic instability and cancer predisposition. Both archaeal Hef and eukaryotic FANCM interact with PCNA, indicating that their role in replication fork is conserved [80]. In *H. volcanii*, it has been shown that *hef* and *hjc* can both be deleted individually but that Hef becomes essential when Hjc is missing [81]. Both single mutants did not have a growth defect, in agreement with our results (Figure 3). However, the single mutants were also not compromised in a homologous recombination assay [81], in stark contrast to the nearly complete loss in gene conversion (Figure 5). This is another indication that in *H. volcanii* intermolecular gene conversion involves enzymes that have function in homologous recombination or other DNA repair pathways (see below) but that the molecular mechanism is separate from these pathways. 

The third single gene with a severe gene conversion defect in the deletion mutant was *nucS*. **NucS (also called EndoMS) is an endonuclease** that acts on both sides of a DNA mismatch, thus generating a double strand break. It has been discovered in thermophilic archaea and is thought to be involved in a non-canonical pathway of DNA mismatch repair (MMS), and thus it is specifically important for species that lack the canonical rep MMS repair pathway involving MutL and MutS. Three recent reviews summarize the characterization of NucS from archaea and from actinobacteria [82,83,84]. Deletion of the *nucS* gene in *Sulfolobus islandicus* led to a 1000-fold increase in the spontaneous mutation rate, underscoring the important role of NucS for genome integrity [85]. Like the two proteins discussed above, Hjc and Hef, NucS also interacts with the replication clamp PCNA. The severe phenotypes of all three mutants indicate that Hjc, Hef, and NucS have important functions in gene conversion and that they are not redundant but all have non-overlapping essential roles.

The **remaining 13 genes with severe gene conversion defects in the respective deletion mutants** were members of several families of paralogous genes. *H. volcanii* and other haloarchaea are characterized by expansions of various genes, which have only a single or very few copies in other archaeal groups. For example, *H. volcanii* contains 12 *orc* (origin recognition complex genes), while *Pyrococcus* has a single *orc* gene [40]. Similarly, *H. volcanii* has four paralogs of TBP (TATA box-binding protein) and twelve paralogs of TFB (transcription factor B), while *Pyrococcus* contains only one copy of these basal transcription initiation factors (www.halolex.mpg.deaccessed at 23 February 2024). In addition, *H. volcanii* contains 18 transcription factors of the Lrp family, while *Pyrococcus* contains one (www.halolex.mpg.de (accessed on 23 February 2024)). Thus, it is not surprising that *H. volcanii* contains several paralogs of Rad3, Rad25, MuL, MutS, and Sph. The gene duplications occurred at different stages during evolution, some of them not before the evolution of the genus *Haloferax* or of the species *H. volcanii* (see Figure 6 and Supplementary Figures S5 and S6). Nevertheless, the drastically reduced gene conversion efficiencies of at least one member in all five families revealed that the functions of the paralogs are not redundant but that novel functions had evolved. 

**MutS, which binds to mismatched DNA, and the nicking endonuclease MutL constitute the canonical mismatch repair pathway**. A phylogenetic analysis revealed that both proteins originated in bacteria and that archaeal homologs were obtained by lateral transfer [86]. Eukaryotes contain a higher number of paralogs, and a recent analysis including Asgard archaea indicated that they were partly obtained from an archaeal progenitor and partly from bacterial endosymbionts [87]. Deletion of the single *mutL* gene in *Halobacterium salinarum* did not increase the mutation rate, which led to the hypothesis that alternative pathways to the canonical MMR exist in haloarchaea [88]. Deletion of both *mutL* paralogs of *H. volcanii* led to a severe decrease in survival, indicating that both are involved in gene conversion (Figure 5) instead of the canonical MMR. However, the picture is complicated by the presence of four paralogs of the MutS family, two each of the subfamilies MutS1 and MutS5. MutS5 from *Pyrococcus horikoshii* has been shown to bind to Holliday junctions [89]. Therefore, it is tempting to speculate that MutS5 is involved in gene conversion while MutS1 is involved in canonical MMR. In agreement with this view, the mutS1a-deletion mutant was not impaired in gene conversion. However, deletion of *mutS1b* also led to a reduced survival in the gene conversion assay (Figure 5). Further experiments are needed to resolve this complex situation of six MutL/MutS paralogs. The bacterial MutL has been shown to be involved in antigenic variation in *B. burgdorfei* [56], indicating that in this species also, MutL has functions beyond MMR. The same is true for the eukaryotic homologs of MutL and MutS, which are called MLH and MSH. They have been shown to be involved in heteroduplex formation, to interact with recombination enzymes, and to promote crossing over during meiosis [90,91,92]. 

Eukaryotic homologs of the archaeal **Rad3 and Rad25 proteins are helicases** involved in nucleotide excision repair (NER) [93]. Their function in archaea is not well studied. The large differences in gene conversion efficiencies (Figure 5) indicates that at least in *H. volcanii*, both Rad proteins are involved in different biological processes.

The last protein family is the **SMC/Sph protein family**. **SMC (Structural Maintenance of Chromosomes)** proteins are highly conserved in archaea, bacteria, and eukaryotes [94]. Eukaryotes have six SMC proteins, SMC1-6. They form three different heterodimers (SMC1-2, SMC3-4, SMC5-6) that build complexes with additional proteins. The SMC protein complexes are involved in DNA condensation, DNA cohesion, and additional genome-related functions [95]. A recent phylogenetic analysis proposed that the six eukaryotic SMC proteins evolved from archaeal SMC proteins via several gene duplication events [96]. Archaea and bacteria typically contain a single SMC protein that forms homodimers and forms functional SMC complexes together with additional proteins. The SMC proteins are comprised of N-terminal and C-terminal globular domains that can bind DNA, two very long coiled-coil domains that are involved in dimerization, and a hinge domain in the middle. In total, SMC proteins have a length of about 1200 aa (amino acids). 

More than 20 years ago, it was discovered that haloarchaea contain proteins that are comprised of the same five domains described above, which were later named **SMC-like proteins of haloarchaea (Sph)** [97]. It was found that heterologous overproduction of a Sph protein from *H. salinarum* or homologous overproduction of a *H. volcanii* Sph in *H. volcanii* results in the formation of very long rods, i.e., that the respective Sphs were involved in cell shape determination. Sequencing of the *H. volcanii* genome revealed that its genome contained four *sph* genes [34]. The Sph protein family is confined to the haloarchaea; however, a recent phylogenetic analysis revealed that several additional subfamilies of SMC-related protein exist [98].

The functions of the Sph proteins remain elusive; however, their SMC-like structure indicate that their globular N- and C-termini might bind DNA and the Sph proteins might be involved in genome-related functions. Therefore, the *smc* gene and all four *sph* genes were included in the present study. The SMC protein is apparently not involved in gene conversion, while, in stark contrast, all four Sph proteins are very important because the respective deletion mutants had severely reduced survival rates of about 10% (Δ*sph1* and Δ*sph3*) or near 0% (Δ*sph2* and Δ*sph4*). Sph2 and Sph4 seem to have overlapping functions, because a double mutant could not be obtained, but also paralog-specific functions based on the very severe phenotype of the single mutants. In a recent study, it was observed that a *sph3*-deletion mutant had lost the ability to form rod-shaped cells [49]. This observation could be verified, and in addition, it was found that the *sph2*-deletion mutant had the opposite phenotype and formed exclusively rod-shaped cells (Supplementary Figure S11). In agreement with earlier reports, the cell shape correlated with the swarming velocity, and Δ*sph3* failed to swarm during the first two days, while Δ*sph 2* was an overswarmer (Supplementary Figure S10). Remarkably, Δ*sph4* behaved exceptionally in this respect, i.e., it had a swarming deficit during the first two days, but the fraction of rod-shaped cells was higher than in the wildtype in exponential phase cultures. Together, these results indicate that the Sph proteins have multiple functions and that they act in the same direction in one biological process (gene conversion) while they have opposing functions in another (cell shape determination). 

The current study used a comprehensive deletion analysis to identify proteins that are involved in intermolecular gene conversion in *H. volcanii*. To deepen the understanding, in **future studies**, alternative approaches should be used to complement the results. For example, co-affinity isolation has been successfully used to unravel the protein–protein interaction network of translation initiation [99], and this approach would be very promising for unravelling the protein–protein interaction network of gene conversion. Notably, this approach would allow researchers to detect the involvement of essential proteins like RadA, which could not be studied with the deletion analysis. It would also unravel whether or not the Sph proteins form specific heterodimers like the eukaryotic SMC proteins. As discussed above, many of the proteins’ specific functions could be predicted, e.g., binding to structured DNA forms or the activity of a helicase or nuclease. The purified proteins could not only be used for the interaction analysis but also to analyze with biochemical assays whether these predictions hold true. 

## 5. Conclusions

The aim of the current study was the identification of proteins that are involved in the molecular mechanisms of the highly efficient intermolecular gene conversion of *H. volcanii*. To this end, 22 single-gene-deletion mutants were generated in two different parent strains. This enabled us to compare the efficiency of gene conversion of the mutants with that of the wildtype in heterozygous cells that were obtained by protoplast fusion. While 6 of the proteins were apparently not involved, 16 mutants were severely deficient in intermolecular gene conversion. Remarkably, mutants of paralogous genes of several gene families had very different phenotypes. The results indicated that the mechanism of gene conversion is separate from homologous recombination, even if several proteins are involved in both processes. For the first time, a biological function of the four members of the Sph protein family could be observed. Identification of the 16 gene conversion proteins paves the way for further experimental approaches to understand the molecular mechanism. 

## Figures and Tables

**Table 1:** List of genes included in this study with protein names and annotated functions.

Protein	Gene	Annotated Function
	HVO_	
Dhfr	∆1279	dihydrofolate reductase (control)
Hjc	∆0170	holliday junction resolvase
Hef	∆3010	ATP-dep. Helicase/nuclease
Hen	∆0418	homing endonuclease
NucS	∆0486	endonuclease
(RadA) ^1^	(0104)	DNA repair + recombination protein
(RadB) ^1^	(2383)	DNA repair + recombination protein
Mre11-Rad50	∆0853/4	double strand break repair protein
Rad3a	∆1351	DNA repair helicase
Rad3b	∆0039	DNA repair helicase
Rad25a	∆A0441	DNA repair helicase
Rad25b	∆1736	DNA repair helicase
Rad25c	∆1598	DNA repair helicase
Rad25d	∆1723	DNA repair helicase
MutLa	∆1939	DNA mismatch repair protein
MutLb	∆0551	DNA mismatch repair protein
MutS1a	∆1940	DNA mismatch repair protein
MutS1b	∆0552	DNA mismatch repair protein
MutS5a	∆0191	DNA mismatch repair protein
MutS5b	∆1354	DNA mismatch repair protein
Smc	∆0689	chromosome segregation protein
Sph1	∆A0180	SMC-like protein
Sph2	∆B0118	SMC-like protein
Sph3	∆2175	SMC-like protein
Sph4	∆B0173	SMC-like protein

^1^ not deleted, essential gene.

## Data Availability

All *H. volcanii* mutant strains generated in this study (or other studies by the Soppa group) are freely available upon request.

## Supplementary Materials

The following supporting information can be downloaded at: https://www.mdpi.com/article/10.3390/genes15070861/s1, Figure S1: dRNA-Seq and RNA-Seq results for *hjc*, *hef*, *hen*, *nucS*; Figure S2: dRNA-Seq and RNA-Seq results for six *rad* genes, Figure S3: dRNA-Seq and RNA-Seq results for six *mut* genes; Figure S4: dRNA-Seq and RNA-Seq results for six *sph*/*smc*/*rad50* genes; Figure S5: Individual neighbor-joining tree of the SMC/Sph protein family, Figure S6: Consensus tree of selected Rad25 proteins, including bootstrap values obtained using three algorithms, Figure S7: Individual neighbor-joining tree of the Rad25 protein family, Figure S8: Consensus tree of selected Mut proteins, including bootstrap values obtained using three algorithms; Figure S9: Individual neighbor-joining tree of the Mut protein family, Figure S10: Swarming of *sph*-deletion mutants and wildtype; Figure S11: cell morphologies of *sph* mutants and wildtype at different optical densities; Table S1: List of all oligonucleotides used for deletion mutant generation; Table S2: Results of the protoplast fusions of the parent strains, the control strains, and the test strains with deletions of genes possibly important for gene conversion.
