# Supplementary material for: Intermolecular Gene Conversion for the Equalization of Genome Copies in the Polyploid Haloarchaeon Haloferax volcanii: Identification of Important Proteins
Source: Genes (Basel). 2024 Jul 1;15(7):861. doi: 10.3390/genes15070861 (PMC11276520; doi:10.3390/genes15070861)
Supplement: Supplementary file 1 [file genes-15-00861-s001.zip › Supplementary Material/Legends_Supplementary _Figures.pdf]

Legends for Supplementary Figures

Suppl. Fig. S1 – S4. Transcript levels of the genes included in this study. Screenshots from the Integrated Genome Browser are shown. The respective genes are shown in the center of the genomic regions, and the names are indicated. Blue arrows indicate annotated protein-coding genes. The results from a dRNA-Seq study are shown in green, which represent transcription start sites. The results from a mixed RNA-Seq study are shown in red, which represent a mixture of the transcript levels of cultures grown under four different conditions (mid-exponential in complex medium, mid-exponential in synthetic glucose medium, mid-exponential in synthetic medium at low salt, stationary phase in complex medium).

Suppl. Fig. S5. Neighbor-joining tree of the SMC/Sph protein family. The neighbor-joining tree was generated with the program MEGA X. The branch lengths represent the average amino acid substitutions per position, the values are shown at each branch, and a scale is given at the bottom of the figure.

Suppl. Fig. S6. Consensus tree of the Rad25 protein family. The tree was generated with the program MEGA X using the maximum likelihood algorithm. Trees were also generated using the maximum parsimony and the neighbor-joining algorithm (not shown). In each case, 1000 bootstrap values were performed, and the bootstrap values (%) are shown at each node. Selected nodes are highlighted by numbers and are discussed in the text.

Suppl. Fig. S7. Neighbor-joining tree of the Rad25 protein family. The neighbor-joining tree was generated with the program MEGA X. The branch lengths represent the average amino acid substitutions per position, the values are shown at each branch, and a scale is given at the bottom of the figure.

Suppl. Fig. S8. Consensus tree of the Mut protein family. The tree was generated with the program MEGA X using the maximum likelihood algorithm. Trees were also generated using the maximum parsimony and the neighbor-joining algorithm (not shown). In each case, 1000 bootstrap values were performed, and the bootstrap values (%) are shown at each node. Selected nodes are highlighted by numbers and are discussed in the text.

Suppl. Fig. S9. Neighbor-joining tree of the Mut protein family. The neighbor-joining tree was generated with the program MEGA X. The branch lengths represent the average amino acid substitutions per position, the values are shown at each branch, and a scale is given at the bottom of the figure.

Suppl. Fig. S10. Swarming velocity of *sph* deletion mutants. The four *in frame sph* deletion mutants and the parent strain (wildtype) were stabbed in the center of swarm plates into the soft agar. At the indicated time points the swarming diameters were measured.

Suppl. Fig. S11. Cell shapes of the *sph* deletion mutants. The four *sph* deletion mutants and the parent strain were grown in complex medium. At early exponential phase (OD_{600} = 0.005), at mid-exponential phase (OD_{600} = 0.3), and during stationary phase (OD_{600} = 1.6) samples were removed and inspected microscopically. Micrographs of all strains at all conditions are shown, strain identity and optical density are shown on top of each figures.
