# Supplementary material for: Intermolecular Gene Conversion for the Equalization of Genome Copies in the Polyploid Haloarchaeon Haloferax volcanii: Identification of Important Proteins
Source: Genes (Basel). 2024 Jul 1;15(7):861. doi: 10.3390/genes15070861 (PMC11276520; doi:10.3390/genes15070861)
Supplement: Supplementary file 1 [file genes-15-00861-s001.zip › Supplementary Material/Supplementary_Table_S1.pdf]

Supplementary Table S1: Overview of oligonucleotides used in this study.

Names and sequences are listed, overhangs are underlined.

Name	Sequence (5‘-3‘)
**M13**	GGAAACAGCTATGACCATG
**M13-20**	GTAAAACGACGGCCAGTG
**P1 *HVO_1279***	GACGGGGCGGTATGGTACGGGACAC
**P4 *HVO_1279***	ACCGGGATGGTGAACGCCGCG
**0104 P1**	GCGACCGCGCCTACTGGATTTAC
**0104 P2**	CGGCGCGTCCACGAGGTCGTAACCGCTCTCGACGAGTTTG
**0104 P3**	GAGAGCGGTTACGACCTCGTGGACGCGCCGAACCTC
**0104 P4**	GCGACTCCGAACGGGACGTACAAG
**2383 P1**	GGACCGCGATGCCCGGTCTC
**2383 P2**	TCTTGAACGTCGCCGGCGCGTCACAGCCGGTGGAG
**2383 P3**	CCGGCTGTGACGCGCCGGCGACGTTCAAGATAACCGACC
**2383 P4**	CGAACATCGGCCTCATCGAAACGC
**3010 P1**	CCGGGCAGTACGAACTCAACCTC
**3010 P2**	GCGATGGCGGAGACGATGAACGACGGCGCGAGTAGC
**3010 P3**	CGCGCCGTCGTTCATCGTCTCCGCCATCGCGGACATC
**3010 P4**	AGCCTCCACGTCGTCCACTACAC
**0170 P1**	GGACGGCGATGCACGAACACG
**0170 P2**	CGAAGTCCTCGCCCTCGCGTCGGTCTCCCTTTCTGTTC
**0170 P3**	AGGGAGACCGACGCGAGGGCGAGGACTTCGAGTCGTTC
**0170 P4**	GCGACGAGACGTTCGTTCCGATG
**0853/4 P1**	GCGCGTGACGCCTTCCAATCG
**0853/4 P2**	CACCGGAGGCTGAGGTGCCGACGATGGCGAGGAAGG
**0853/4 P3**	TCGCCATCGTCGGCACCTCAGCCTCCGGTGTGCAATCTAC
**0853/4 P4**	GAGCGACCTCCACGGTCTCTTG
**1939 P1**	GCTCGCGGCCTACGTCGATGG
**1939 P2**	CGGTACGTTCCGGTTGCGGCGATACTCGGGAGTGC
**1939 P3**	CCCGAGTATCGCCGCAACCGGAACGTACCGCGTCGAAG
**1939 P4**	TGGCTCGACGGCGACGAGTACG
**0551 P1**	GGCCGTCGAAGGGAGTGCTG
**0551 P2**	CGCAGTCGTCGAGCGCCGCGCCGATGGTGTGGAGC
**0551 P3**	ACACCATCGGCGCGGCGCTCGACGACTGCGAGAACC
**0551 P4**	ACGGGCGAGGGAACCCAGTC

**1351 P1**	ACACTCGCCACCATCTCGGGAAC
**1351 P2**	GAGTCGCGGGCGTACACGAAGACGCGCTCGTAGTCGG
**1351 P3**	CGAGCGCGTCTTCGTGTACGCCCGCGACTCGTGGAAC
**1351 P4**	CGGACATTCGCCGTCACGTACAC
**P1 HVO_0191**	CATCGTCCGGCAGTGGTAAC
**P2 HVO_0191**	GTCCGACGGCCTCGAGCTCGGCGTCGTCGAGTTC
**P3 HVO_0191**	TCGACGACGCCGAGCTCGAGGCCGTCGGACTGG
**P4 HVO_0191**	TCGAGCGTGACGGGAACTAC
**Ana HVO_0191 Del fw**	CGTCGTCCCGGAGTCACCAC
**Ana HVO_0191 Del rv**	CAGCAGTCGCCCGTAGAAACTCG
**P1 HVO_1736**	CCTCGGCTGTCGTCTGATCG
**P2 HVO_1736**	GCGAGCATGGAGTACGGAACTGTCTCGGGCGCATC
**P3 HVO_1736**	GCCCGAGACAGTTCCGTACTCCATGCTCGCCACCTC
**P4 HVO_1736**	GTCGTTGTCGTCGTGGACTC
**Ana HVO_1736 Del fw**	GCTGTGCCGTCACTGGGTGC
**Ana HVO_1736 Del rv**	TCGTAGAGCCGTGCTGGCTG
**P1 HVO_0418**	CCCGACCGCTGCGAGGTGAC
**P2 HVO_0418**	AGAGGGACGATATGGCGGCGTGCTGTCGACTCGTAGG
**P3 HVO_0418**	GTCGACAGCACGCCGCCATATCGTCCCTCTCGGTGAGCC
**P4 HVO_0418**	TCGCGGCGGAGTTCGCGCAG
**Ana_FW_HVO_0418**	AAATTCACCGTCCGTCCTTC
**Ana_RV_HVO_0418**	TCCACGGCTAACTGTCTATC
**P1 HVO_1723**	CGCTCGTGATGGCGAAGCTC
**P2 HVO_1723**	GCCGACTCAGCACCGTCGGTTACGTCGGCCAATCG
**P3 HVO_1723**	GGCCGACGTAACCGACGGTGCTGAGTCGGCGTCTG
**P4 HVO_1723**	CGGGCCGCTAACTGTCGAAC
**Ana_FW_HVO_1723**	GTATTCGGCTTCGATGACTG
**Ana_RV_HVO_1723**	GAGCTAAGAGCGGTCAAGAG
**P1 HVO_0039**	CAGAGGACGACGCCGTAGCC
**P2 HVO_0039**	GTCGTGGTCGGTGTACGCCTCCTGATTCGGGTACG
**P3 HVO_0039**	CCGAATCAGGAGGCGTACACCGACCACGACGCCTAC
**P4 HVO_0039**	CGGTCATCATGCCGGCCATC
**Ana HVO_0039 fw**	ATTATACCGGACCTGTCGAG
**Ana HVO_0039 rev**	GCACTCATCACCCTGTGTTG
**P1 HVO_1940**	TCGGGCGTGAACTGAATGCG
**P2 HVO_1940**	CCACGAGGTCGGCCTCTCGCACTGCCAGCATCTCC

**P3 HVO_1940**	TGCTGGCAGTGCGAGAGGCCGACCTCGTGGACACC
**P4 HVO_1940**	TCGGCAAACAGGCCGGTCAC
**Ana HVO_1940 fw**	GTACATTGGCCTTGCTGGTC
**Ana HVO_1940 rev**	GTCGGTGAATCATTCGAGTC
**P1 HVO_1354**	GGGTGTTTCGTTCGTGTCTC
**P2 HVO_1354**	ACCGCTTCTTCGCCCCCGAGCGCCGATTCCAGTC
**P3 HVO_1354**	GGAATCGGCGCTCGGGGGCGAAGAAGCGGTACAG
**P4 HVO_1354**	CCGACGCAACGAAACTGTG
**Ana HVO_1354 fw**	GAACAGCGGCGGCAC
**Ana HVO_1354 rv**	CGACGCGGTCGATGC
**P1 HVO_1598**	CCGTACATCGCCCGCATC
**P2 HVO_1598**	TCGTAGAGCGTCGCGCTGTCGCCGTCAGTGCCG
**P3 HVO_1598**	CACTGACGGCGACAGCGCGACGCTCTACGAACTC
**P4 HVO_1598**	CGCGACGGTGAGTGAGAC
**Ana HVO_1598 fw**	GGAACCCCGAGACACTCC
**Ana HVO_1598 rv**	ACGCCTTCTCCGACGAC
**P1 HVO_A0441**	GGTATGCCACGAGCCGAGAG
**P2 HVO_A0441**	CCCTCGTCGAGAACCTCCGCATACGGGGTATCGTC
**P3 HVO_A0441**	TACCCCGTATGCGGAGGTTCTCGACGAGGGGATTG
**P4 HVO_A0441**	TCACCGGTCCTCTCGTATCG
**P1 HVO_0552**	CGTGTCCACGGTGGTCAG
**P2 HVO_0552**	TTCCGTCTCCGGGTCCTGCATGGCGAGCAGGTC
**P3 HVO_0552**	CTGCTCGCCATGCAGGACCCGGAGACGGAAGCC
**P4 HVO_0552**	TCGCCGTCGTCTTGAGGAAC
**Ana HVO_0552_fw**	ATCACACGCCACATCGAG
**Ana HVO_0552_rv**	TCGTCGAGCTGTTTGATG
**P1 HVO_0689**	CCCGGAACTCTCCTCACACG
**P2 HVO_0689**	CGCCGAACTGGATACCGTAGAACGGGATGCGAGTC
**P3 HVO_0689**	GCATCCCGTTCTACGGTATCCAGTTCGGCGGCGAC
**P4 HVO_0689**	AGGAACGCGTCCGTGACCTC
**ANA_HVO_0689_fw**	GCCCGCGTGAACACCAACCC
**ANA_HVO_0689_rv**	GCCGGCGAACGAGAAGACCC
**P1 HVO_2175**	GATGAGGCCGGCGCACAC
**P2 HVO_2175**	GAGGAACGTCGGGTAGACGCGCCGCTCGTCGATAC
**P3 HVO_2175**	GACGAGCGGCGCGTCTACCCGACGTTCCTCGTCGC
**P4 HVO_2175**	CCGCGGGCGTGTTTGTCC

**ANA_HVO_2175_fw**	CGAGTAGCGATTCGGGCTCACC
**ANA_HVO_2175_rv**	CGAAGCGGAGACGCGGGAGAC
**P1HVO_B0118**	AAGGCGAGGTCGAGAACCTG
**P2HVO_B0118**	CGCGAAGTAGTCCACGCCACCGATATTCTCGACCGAAAG
**P3HVO_B0118**	GAGAATATCGGTGGCGTGGACTACTTCGCGGACTAC
**P4HVO_B0118**	CTCGACGGCGAGCGGGAAAG
**P1 HVO_0486**	TACCACGCGTTGTCTTCGAG
**P2 HVO_0486#2**	CTCGCTCTCTTCGGCGGCTTCGAGGTGGACGAGG
**P3 HVO_0486#2**	GTCCACCTCGAAGCCGCCGAAGAGAGCGAGAAAGC
**P4 HVO_0486#2**	ACGCTCGGCGTCTGTTTC
**P1 HVO_B0173**	CGTCCACTGCCCGGACTGTAAC
**P2 HVO_B0173**	CCGGACACGTCAACGTCGTCGAAGCCGCCGATTCC
**P3 HVO_B0173**	CGGCGGCTTCGACGACGTTGACGTGTCCGGCGAGAG
**P4 HVO_B0173**	GCGCTGCACCGTCGGTACAC
**P1 HVO_A0180**	GACATCGACGCCGACGAGGTG
**P2 NEW HVO_A0180**	GTCGCCGTCCCGGAGCGAATCCCGGCGACGTGCTC
**P3 NEW HVO_A0180**	CGTCGCCGGGATTCGCTCCGGGACGGCGACGTATC
**P4 HVO_A0180**	TGCCGGTCGAGCACGAAGTC
