# Supplementary figures and images for: Intermolecular Gene Conversion for the Equalization of Genome Copies in the Polyploid Haloarchaeon Haloferax volcanii: Identification of Important Proteins
Source: Genes (Basel). 2024 Jul 1;15(7):861. doi: 10.3390/genes15070861 (PMC11276520; doi:10.3390/genes15070861)

# Suppl. Fig. S5

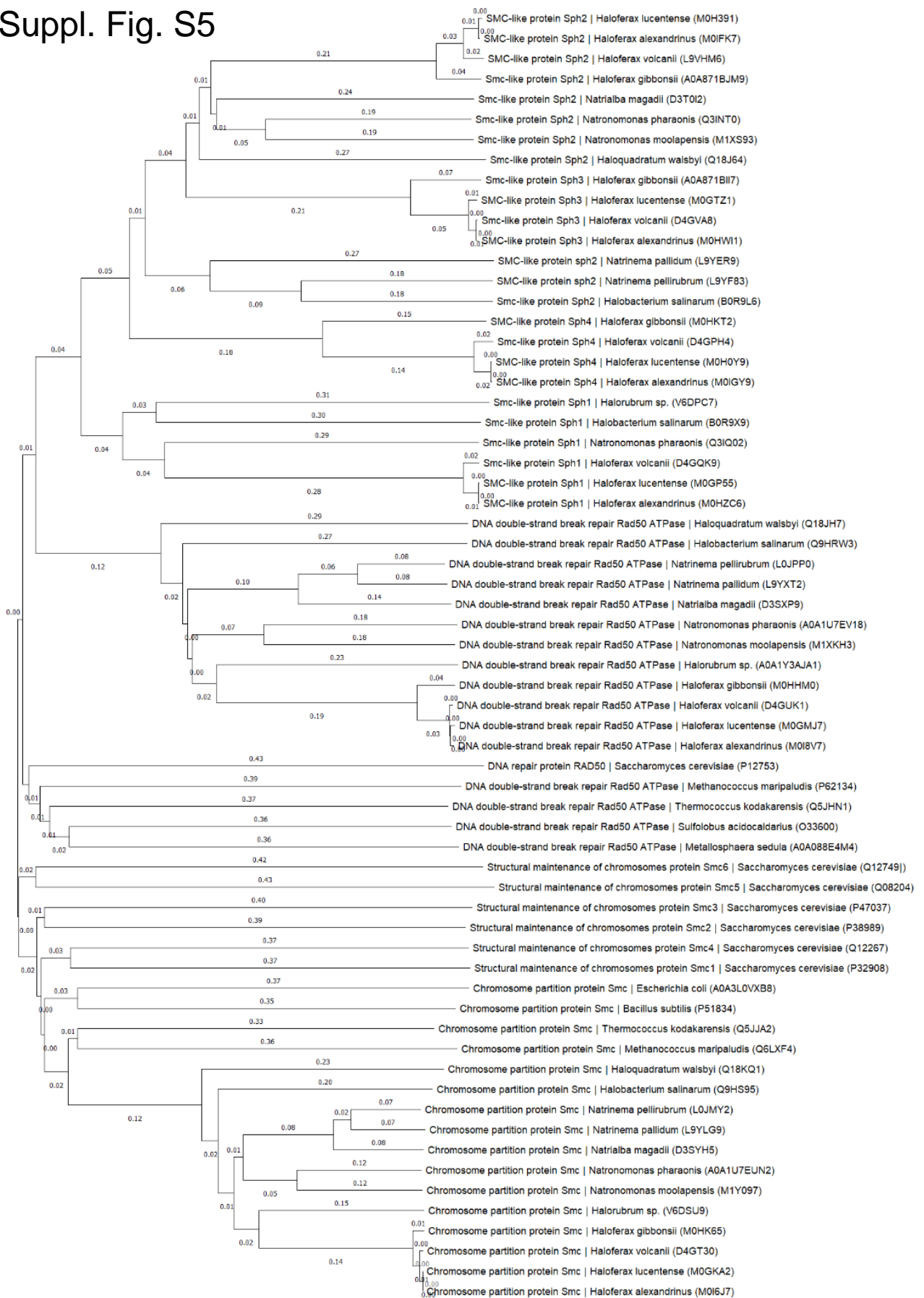

Supplement: Supplementary file 1 [file genes-15-00861-s001.zip › Supplementary Material/Supplementary_Figure_S5.pdf]

# Suppl. Fig. S6

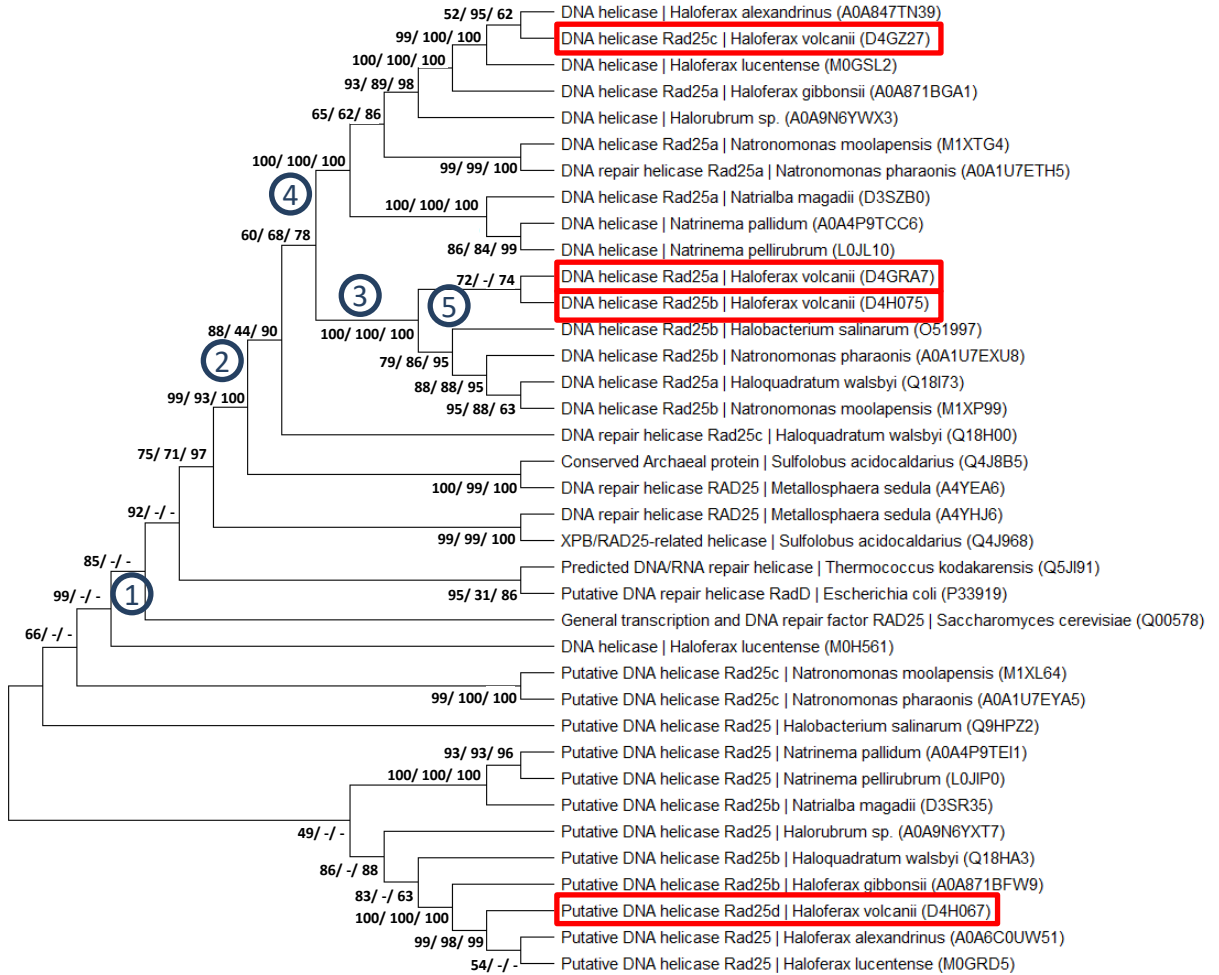

Supplement: Supplementary file 1 [file genes-15-00861-s001.zip › Supplementary Material/Supplementary_Figure_S6.pdf]

Suppl. Fig. S7

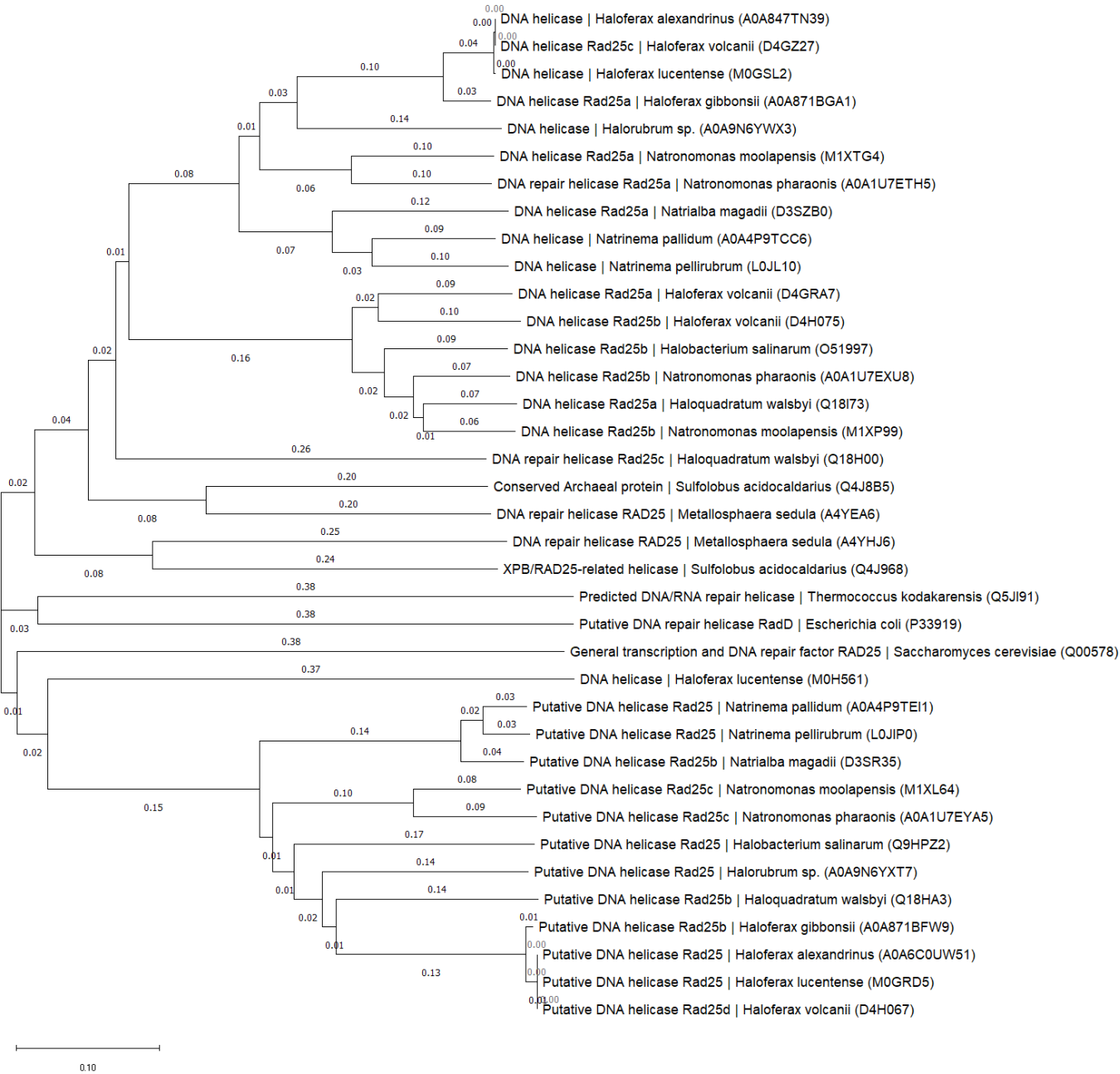

Supplement: Supplementary file 1 [file genes-15-00861-s001.zip › Supplementary Material/Supplementary_Figure_S7.pdf]

# Suppl. Fig. S8

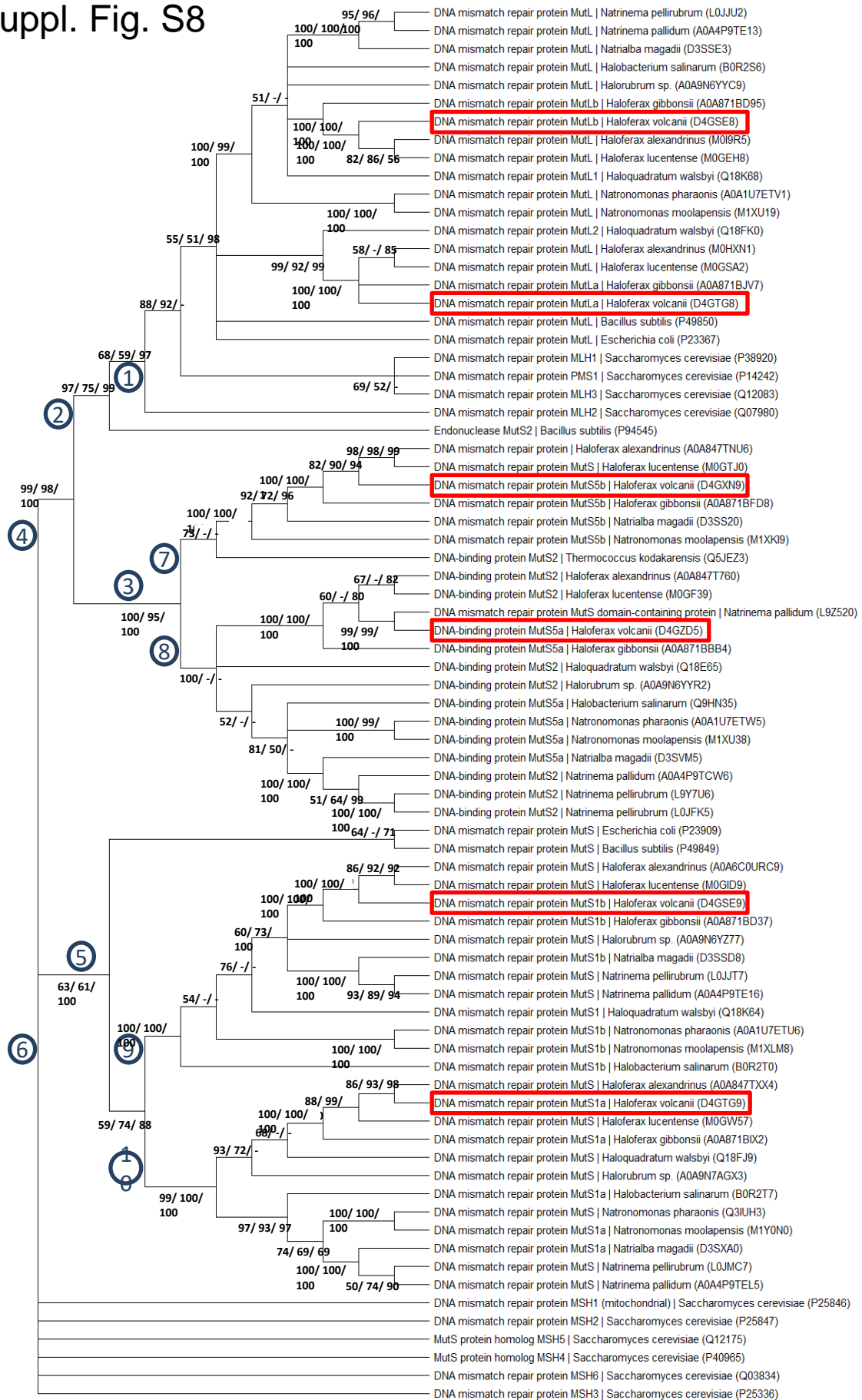

Supplement: Supplementary file 1 [file genes-15-00861-s001.zip › Supplementary Material/Supplementary_Figure_S8.pdf]

# Suppl. Fig. S9

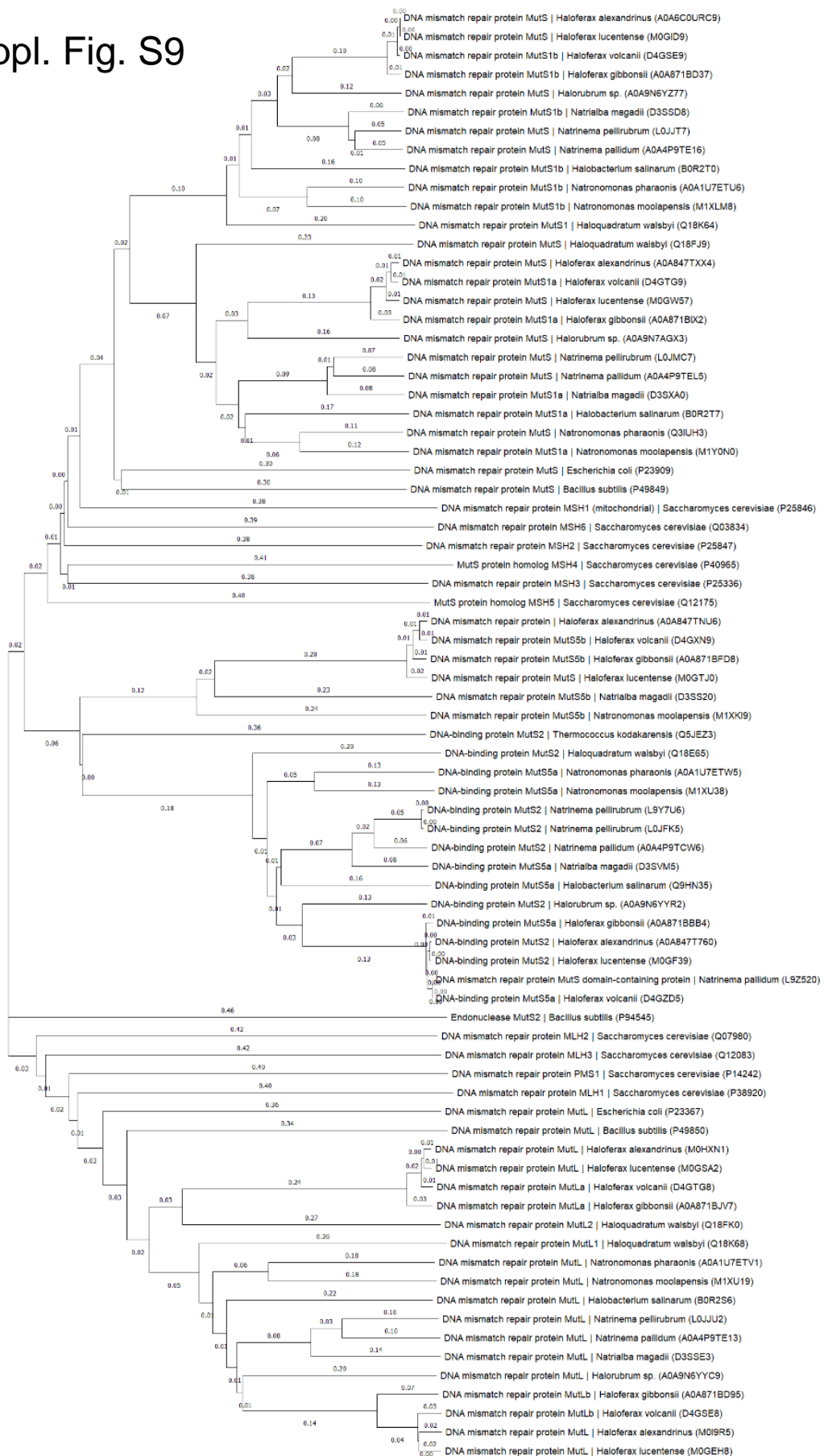

Supplement: Supplementary file 1 [file genes-15-00861-s001.zip › Supplementary Material/Supplementary_Figure_S9.pdf]

Supplementary Figure S10

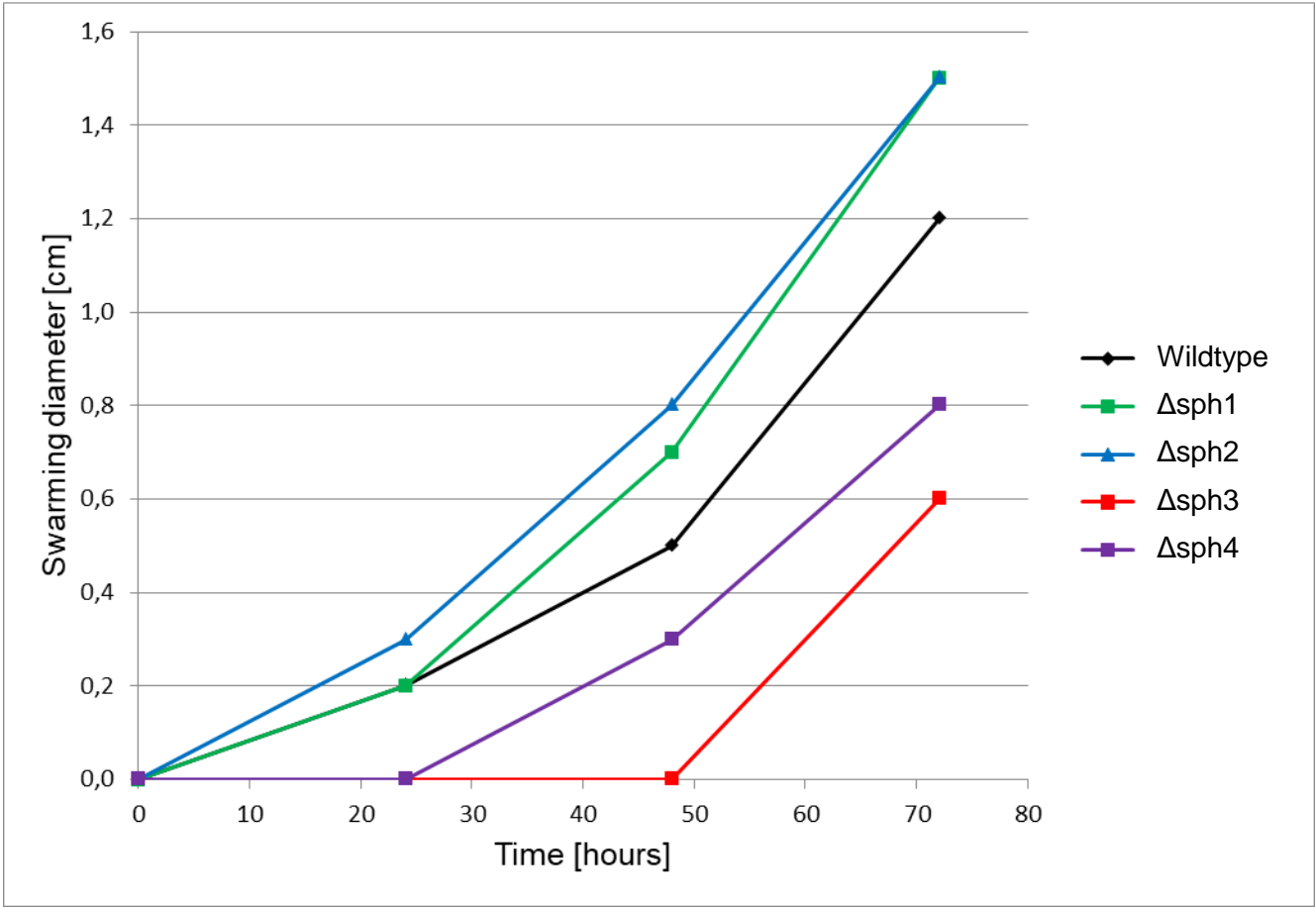

Supplement: Supplementary file 1 [file genes-15-00861-s001.zip › Supplementary Material/Supplementary_Figure_S10.pdf]

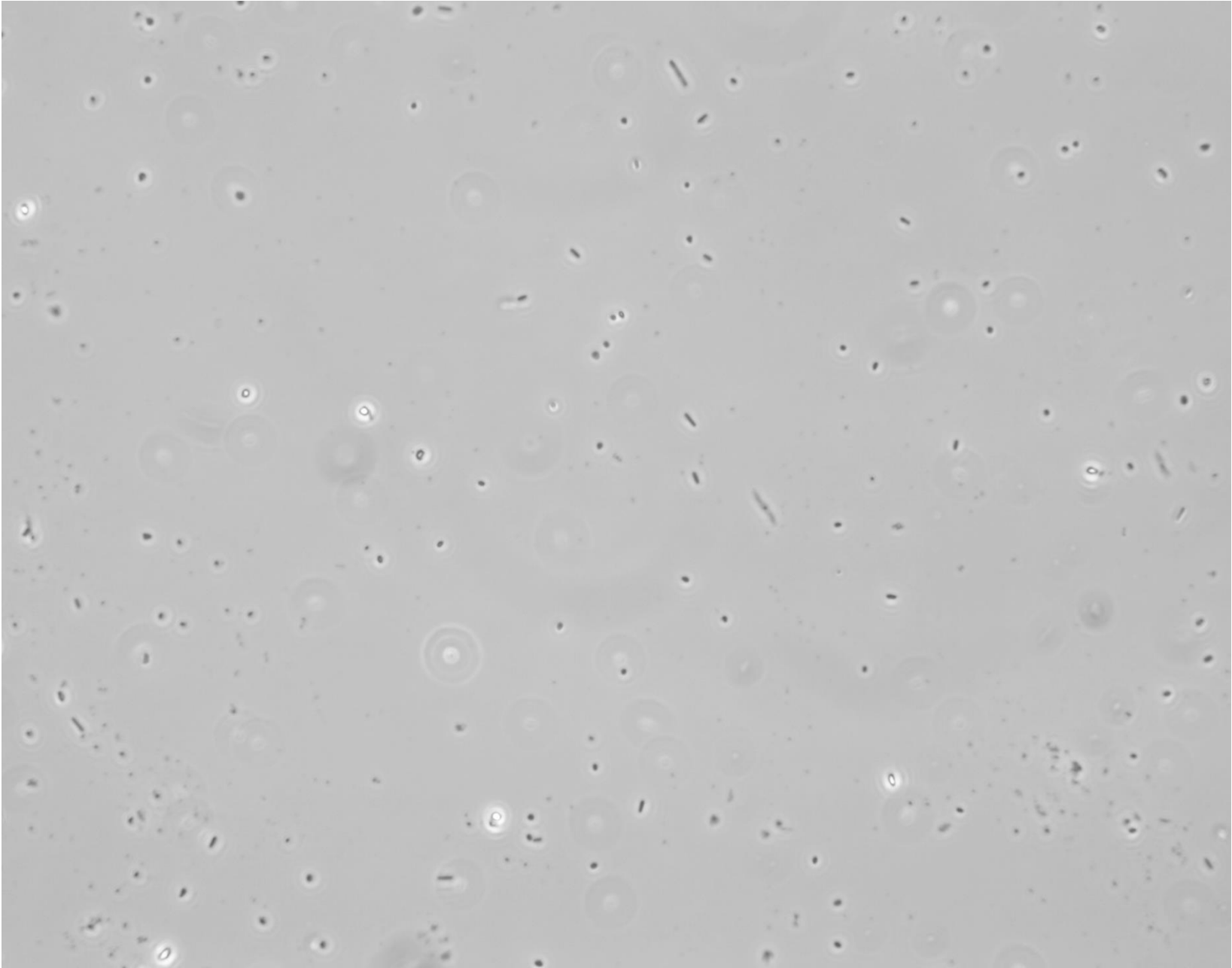

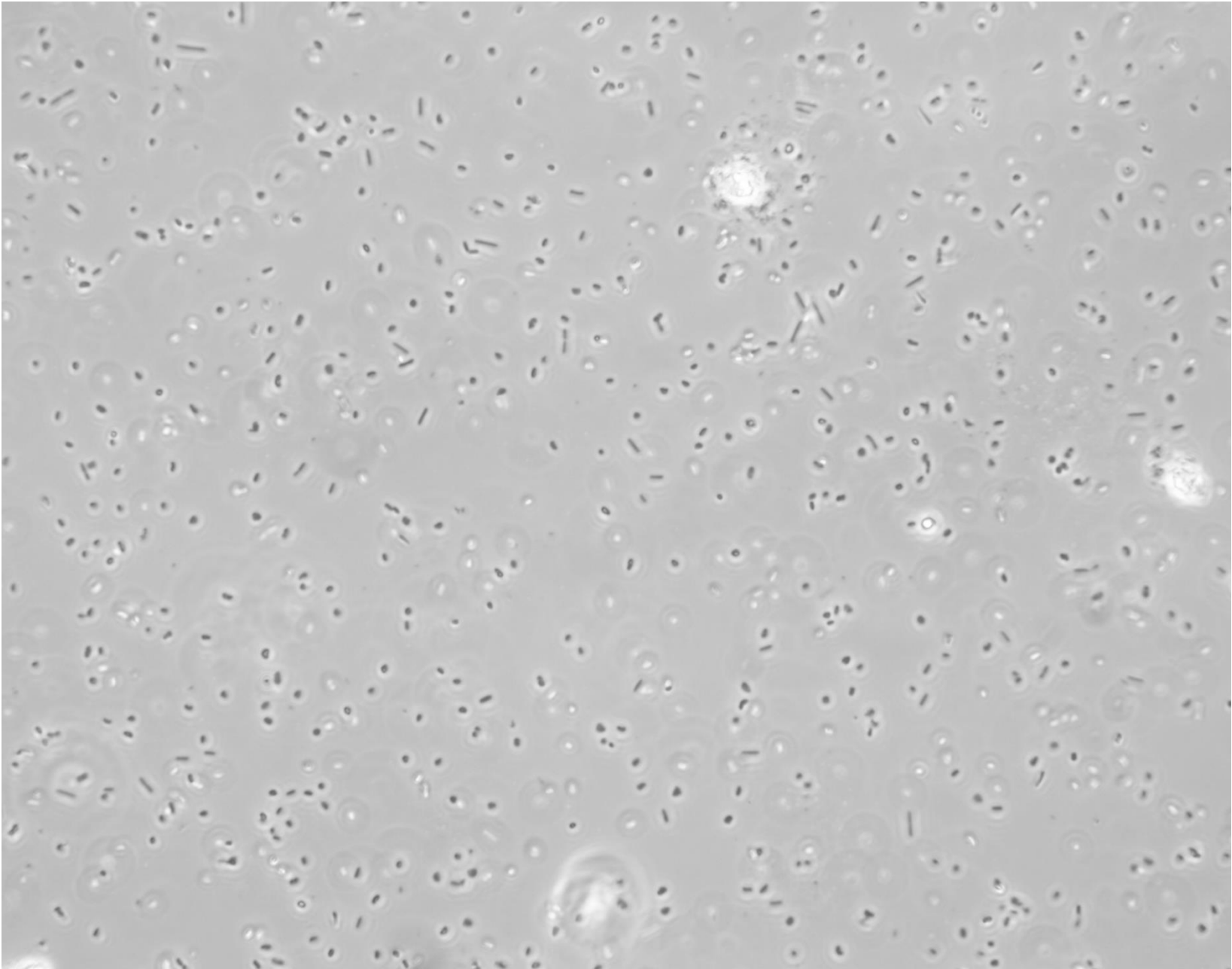

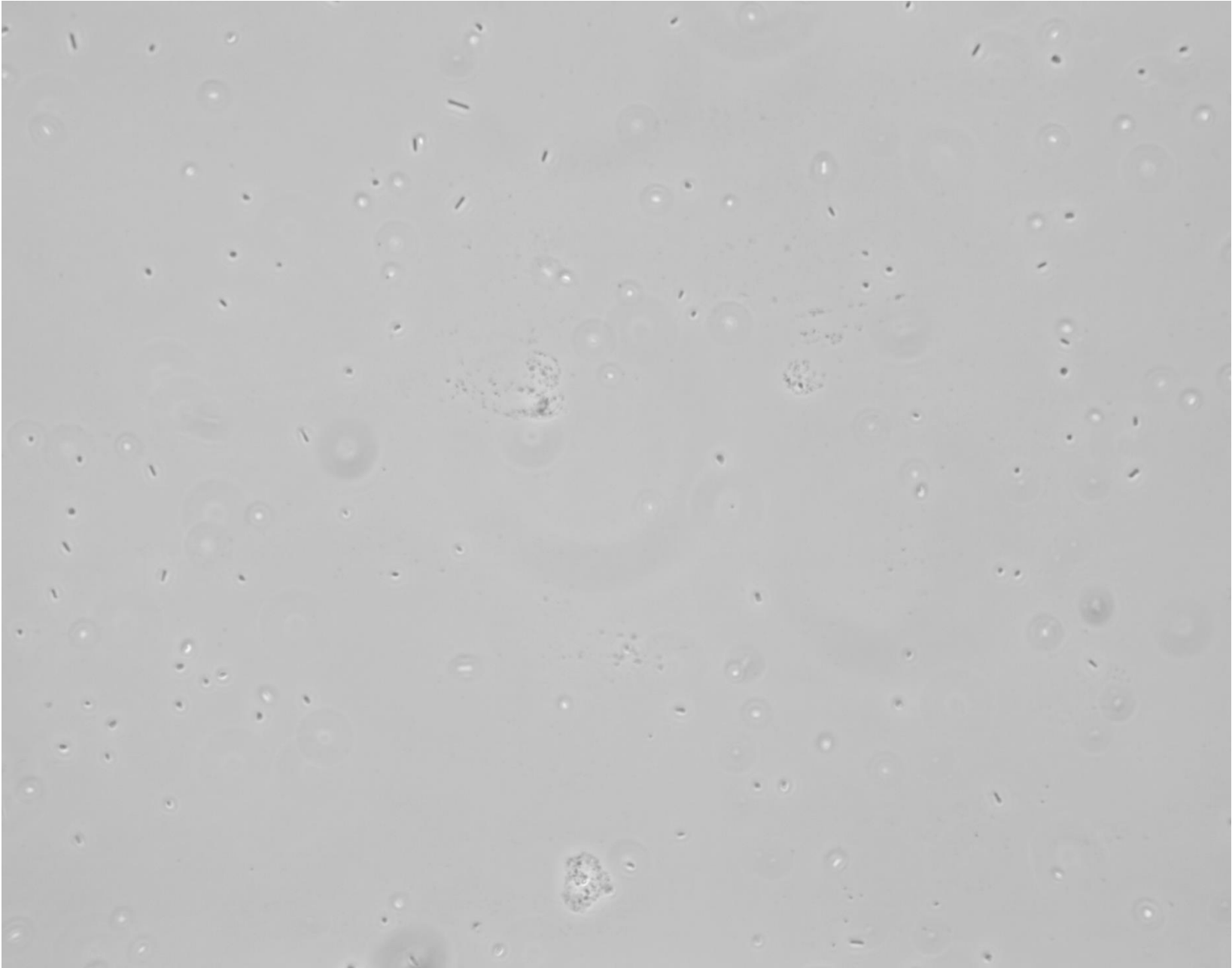

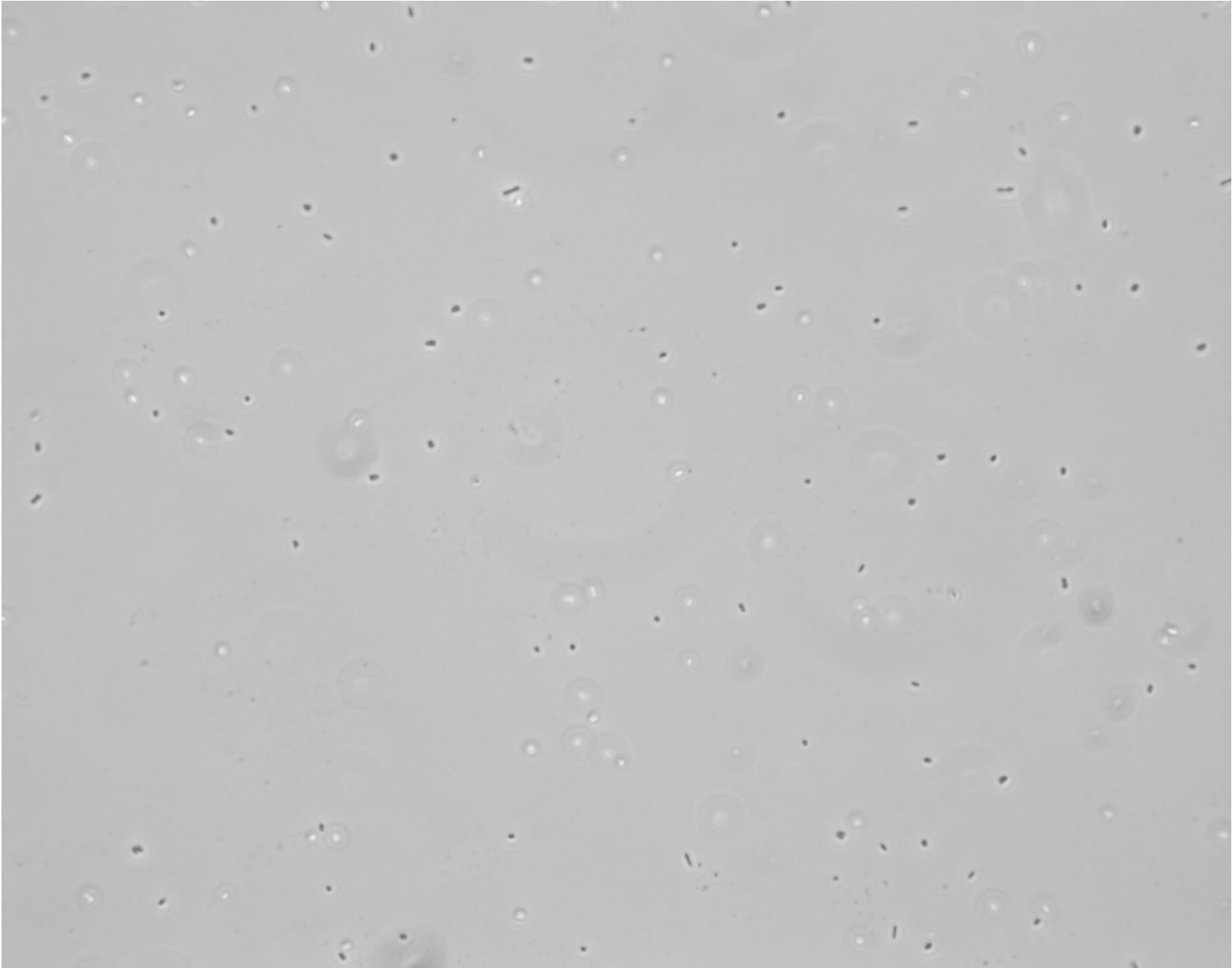

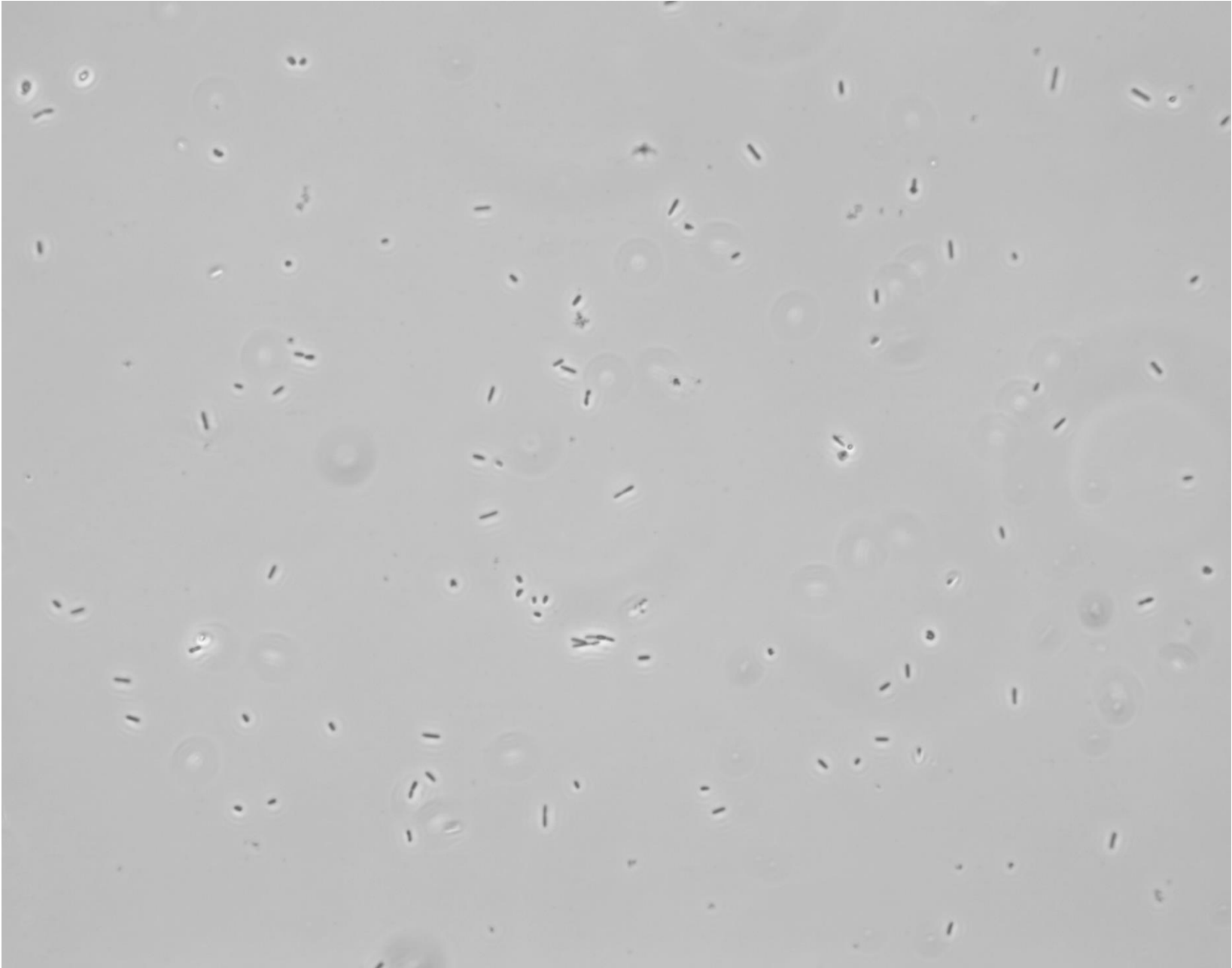

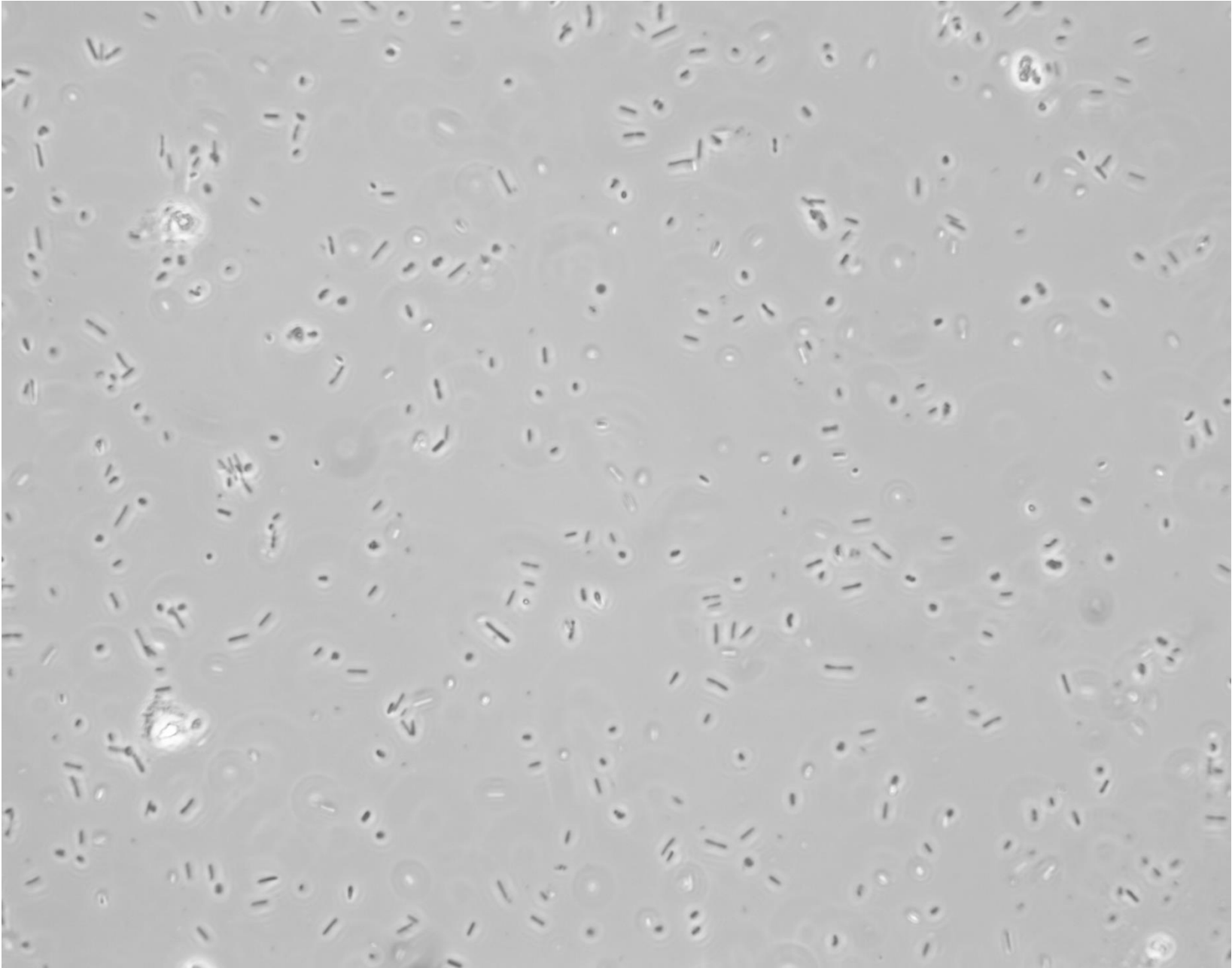

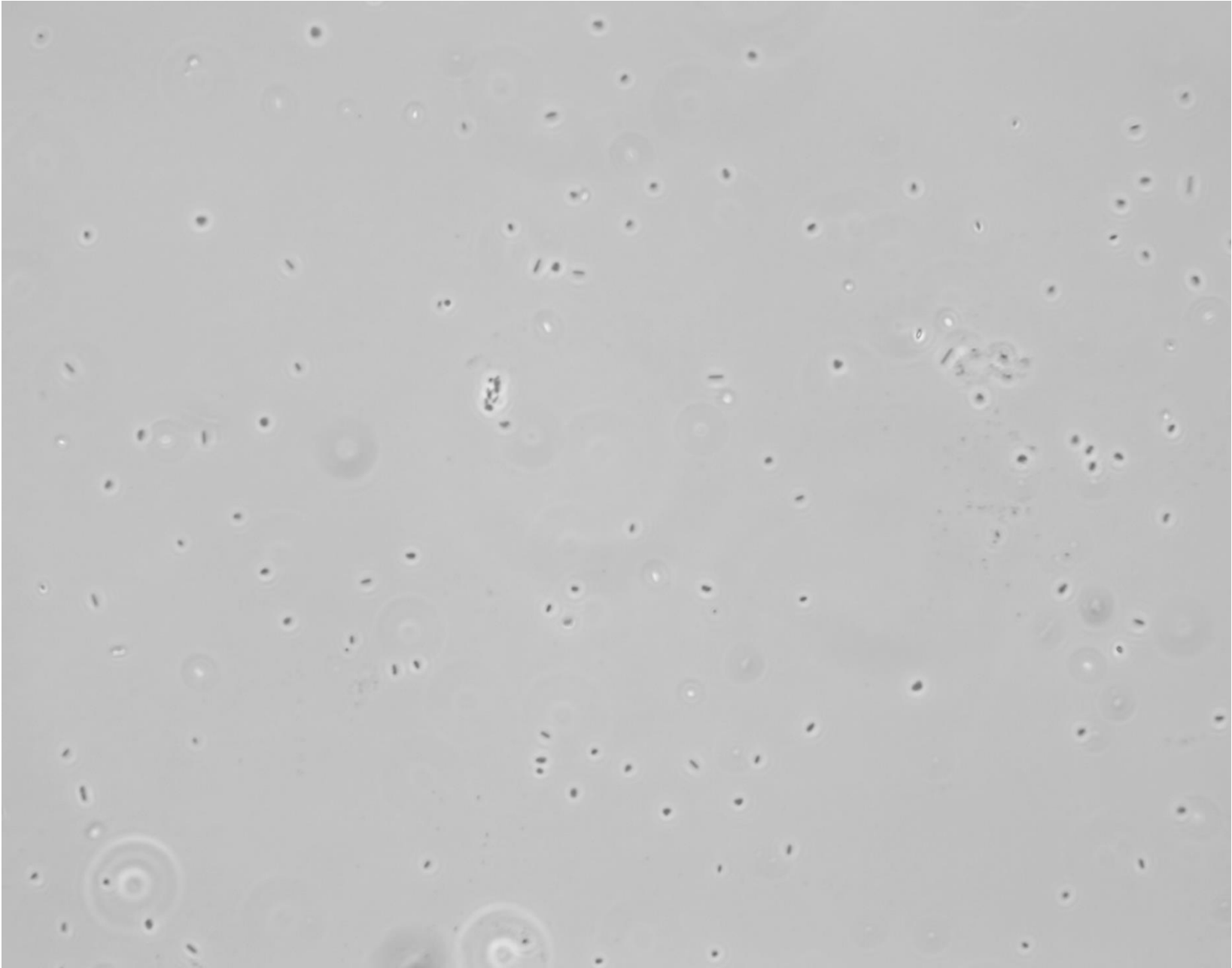

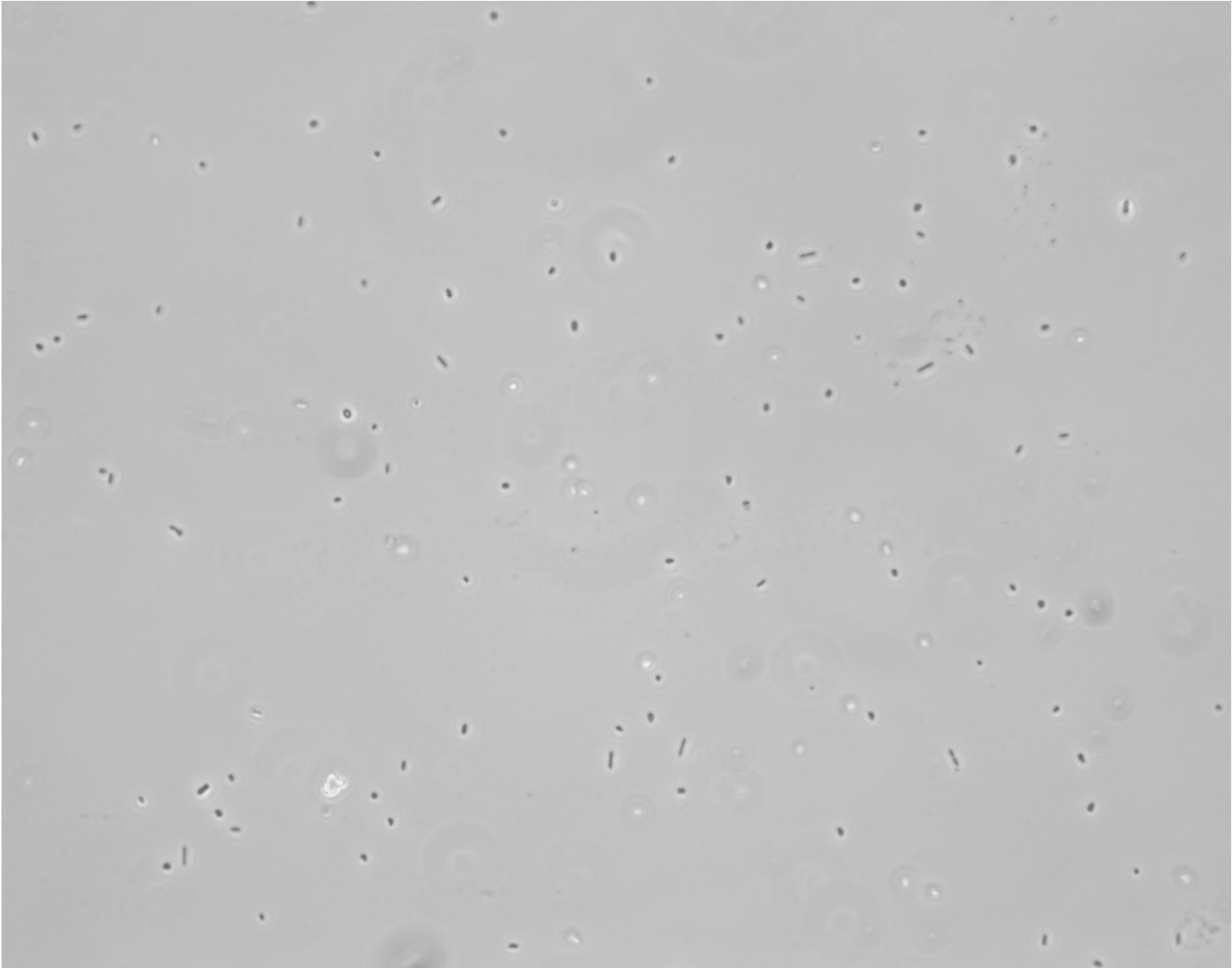

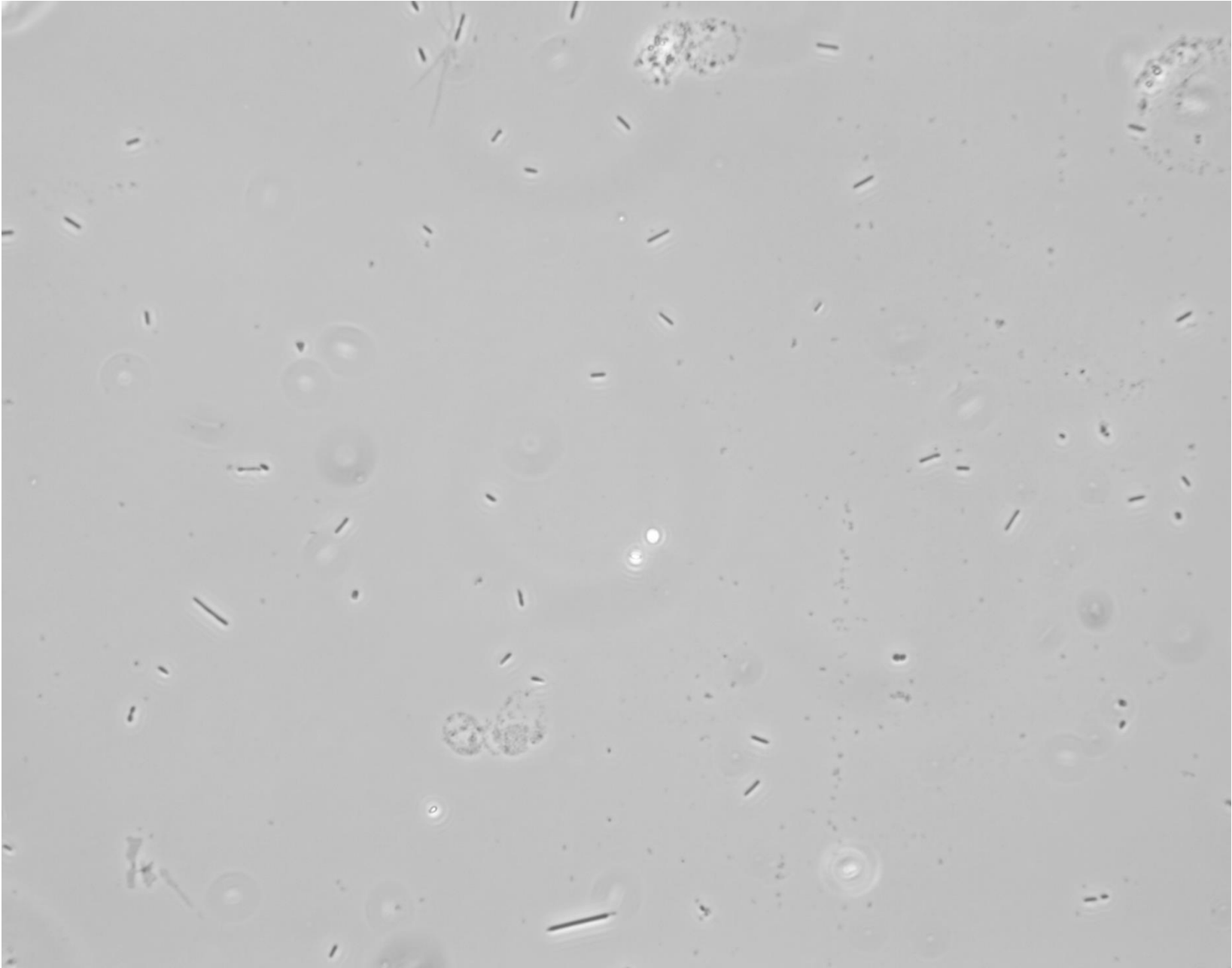

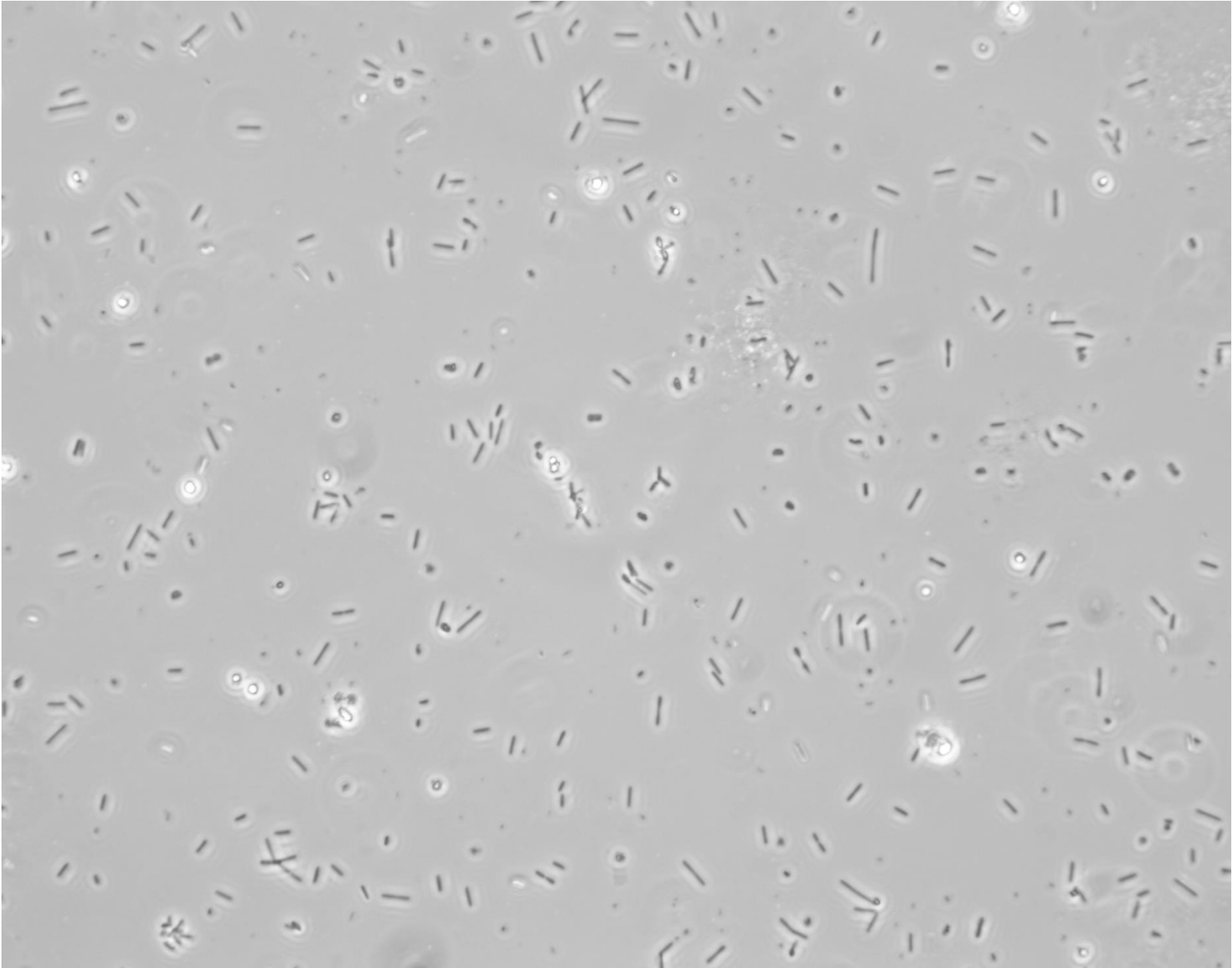

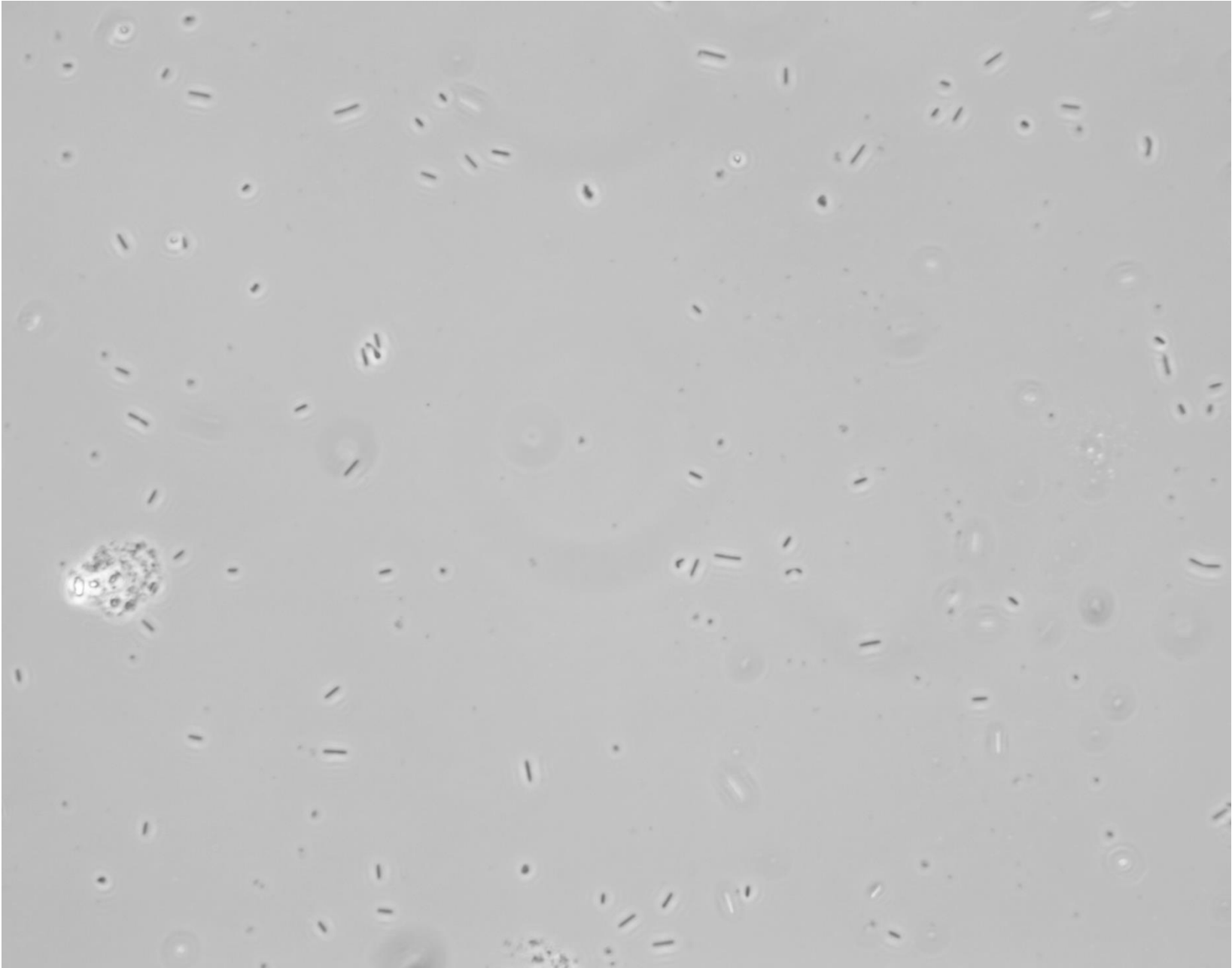

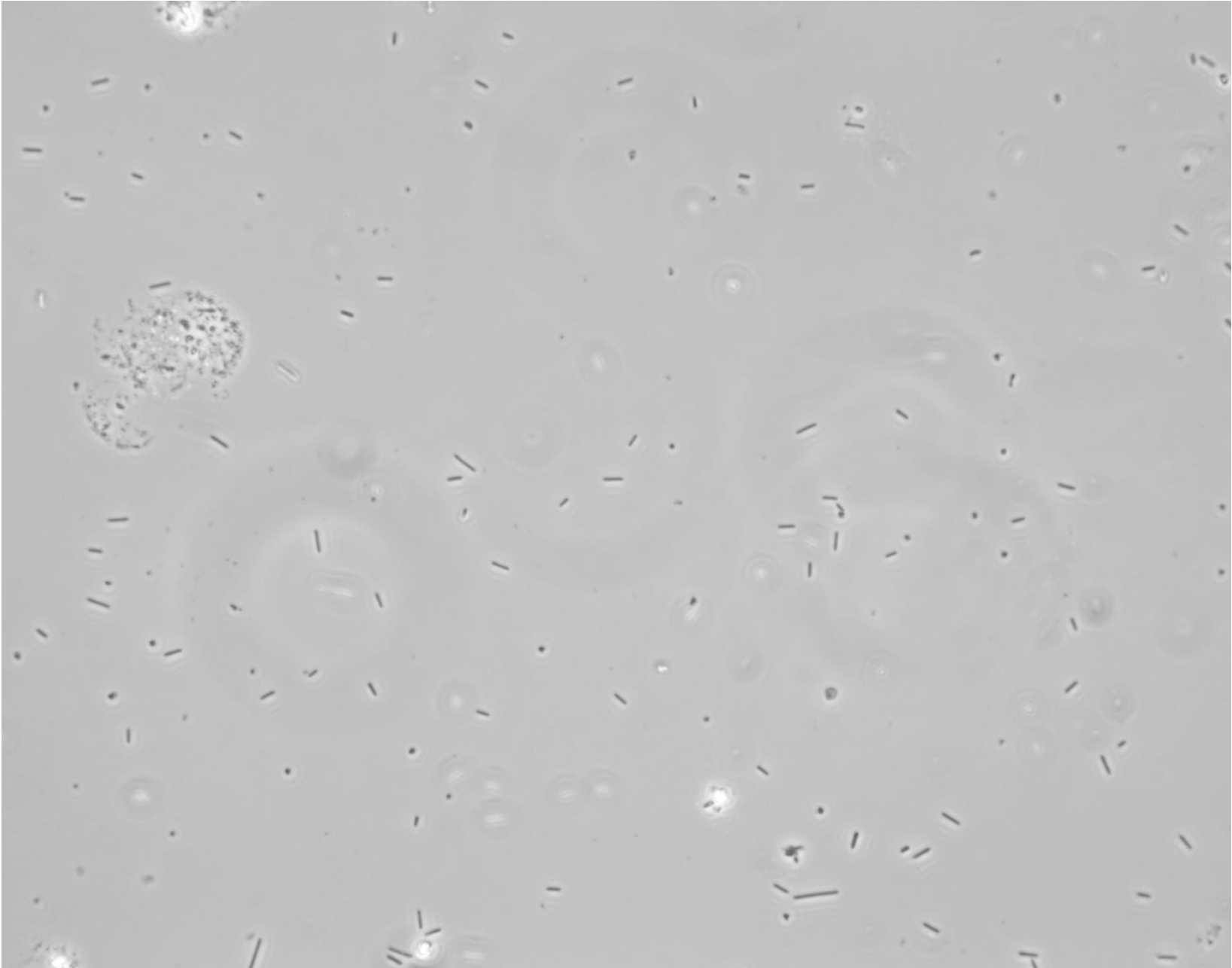

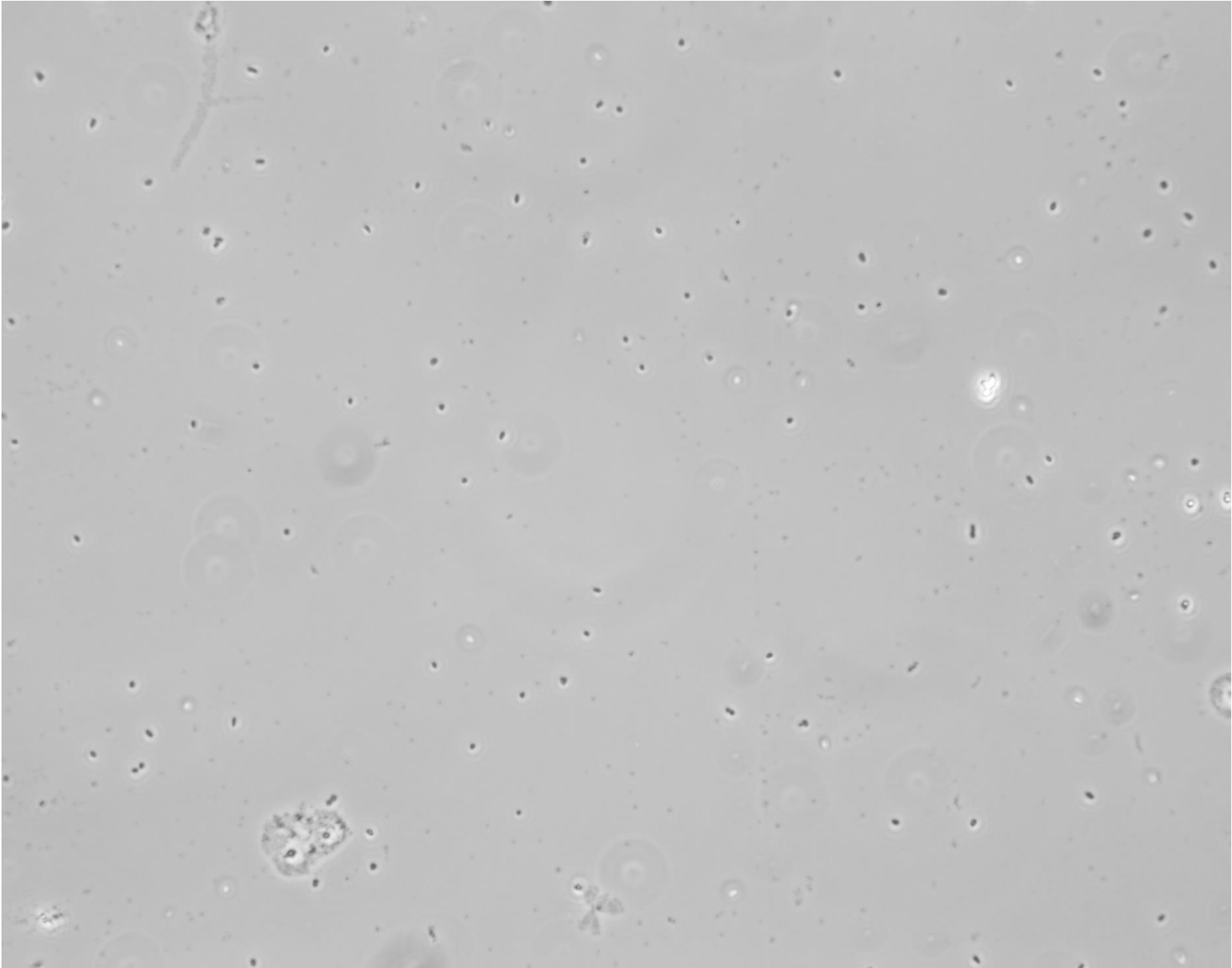

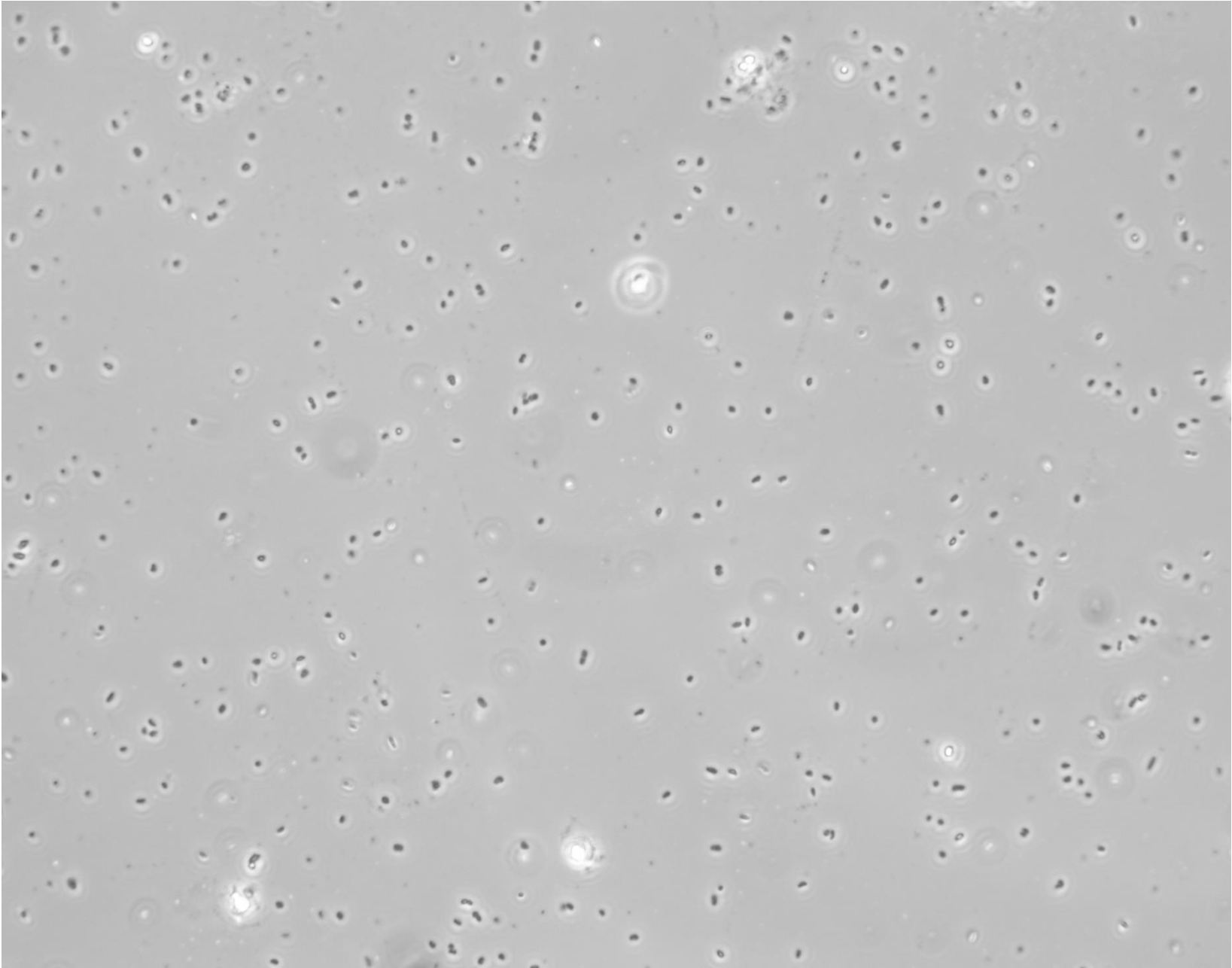

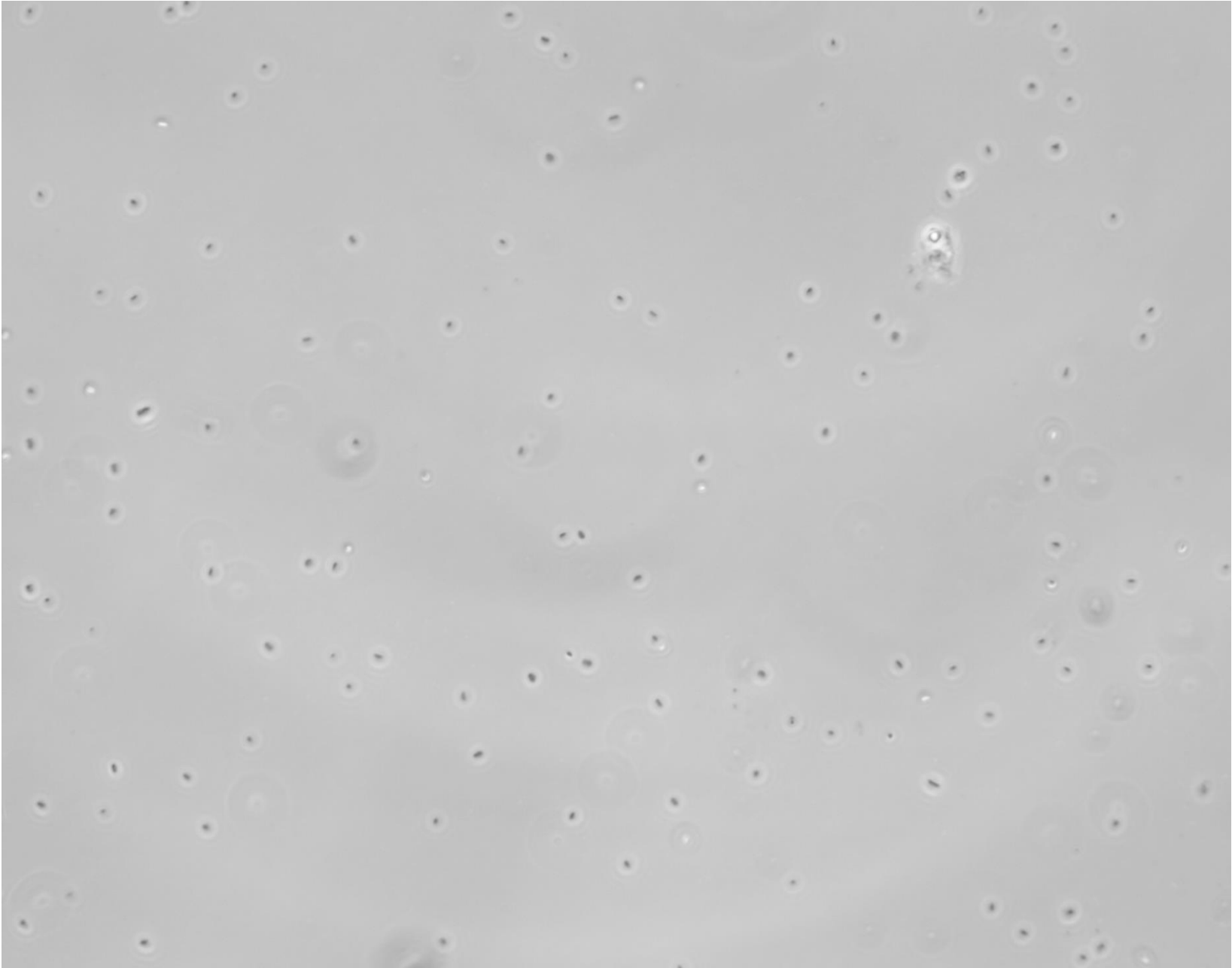

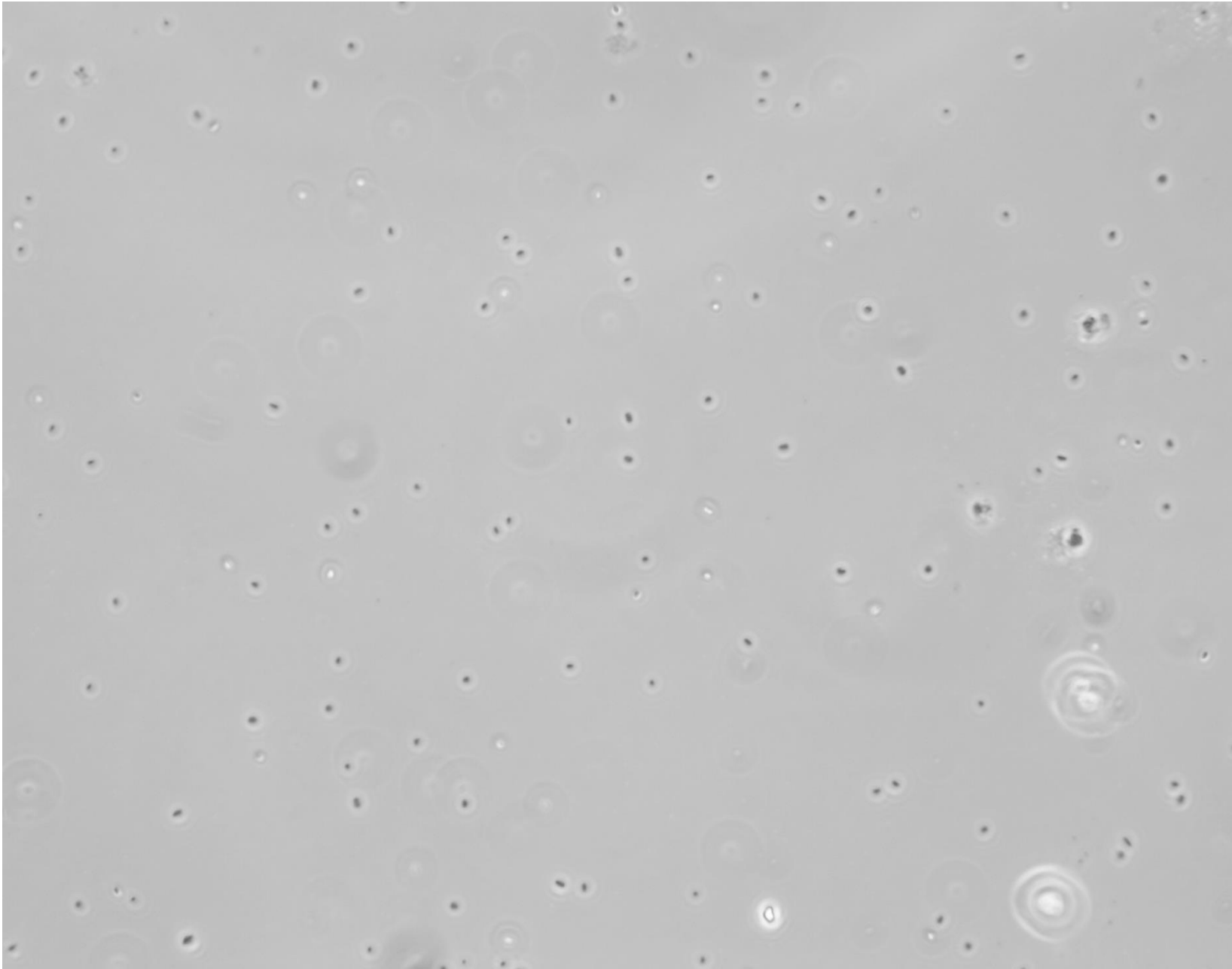

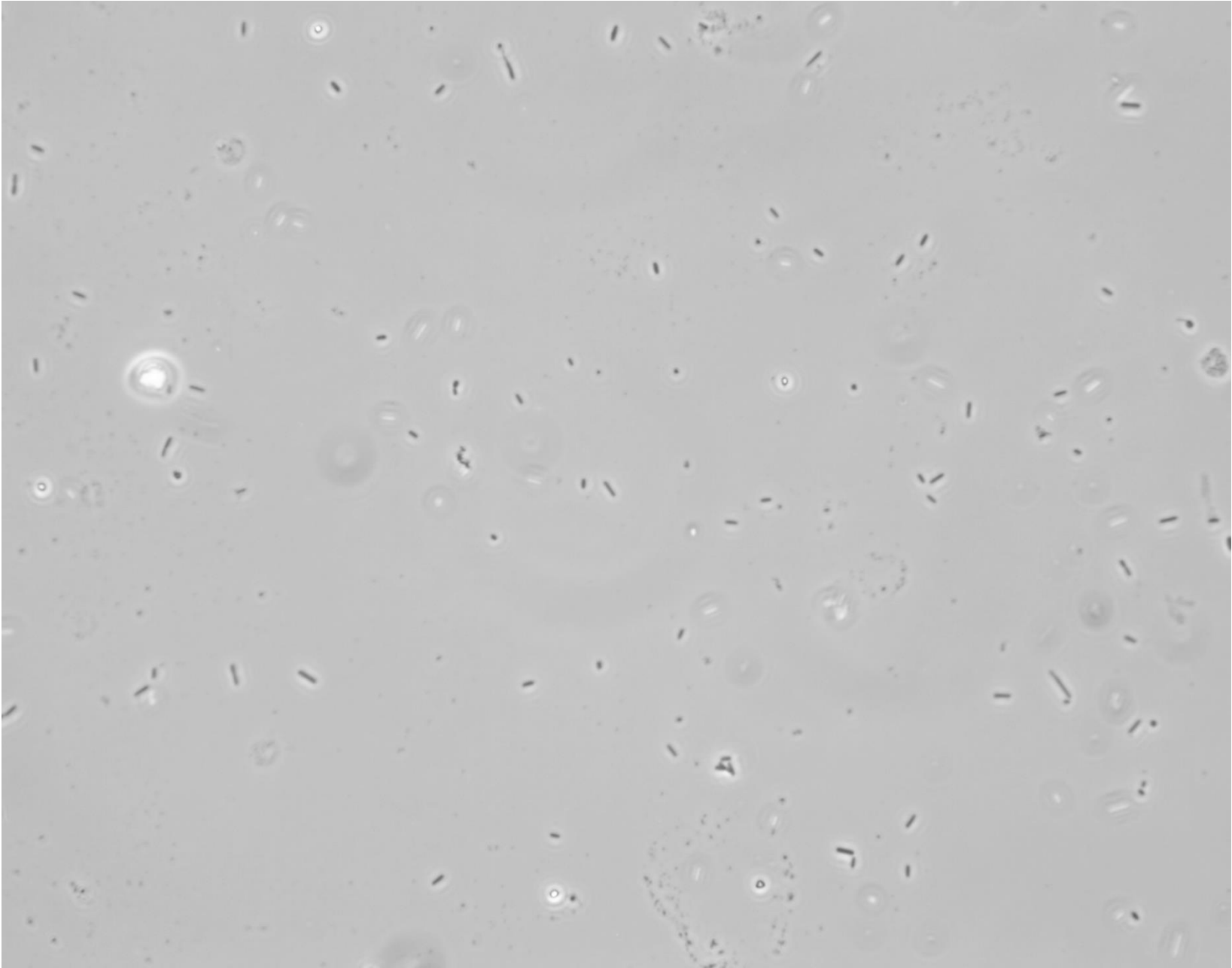

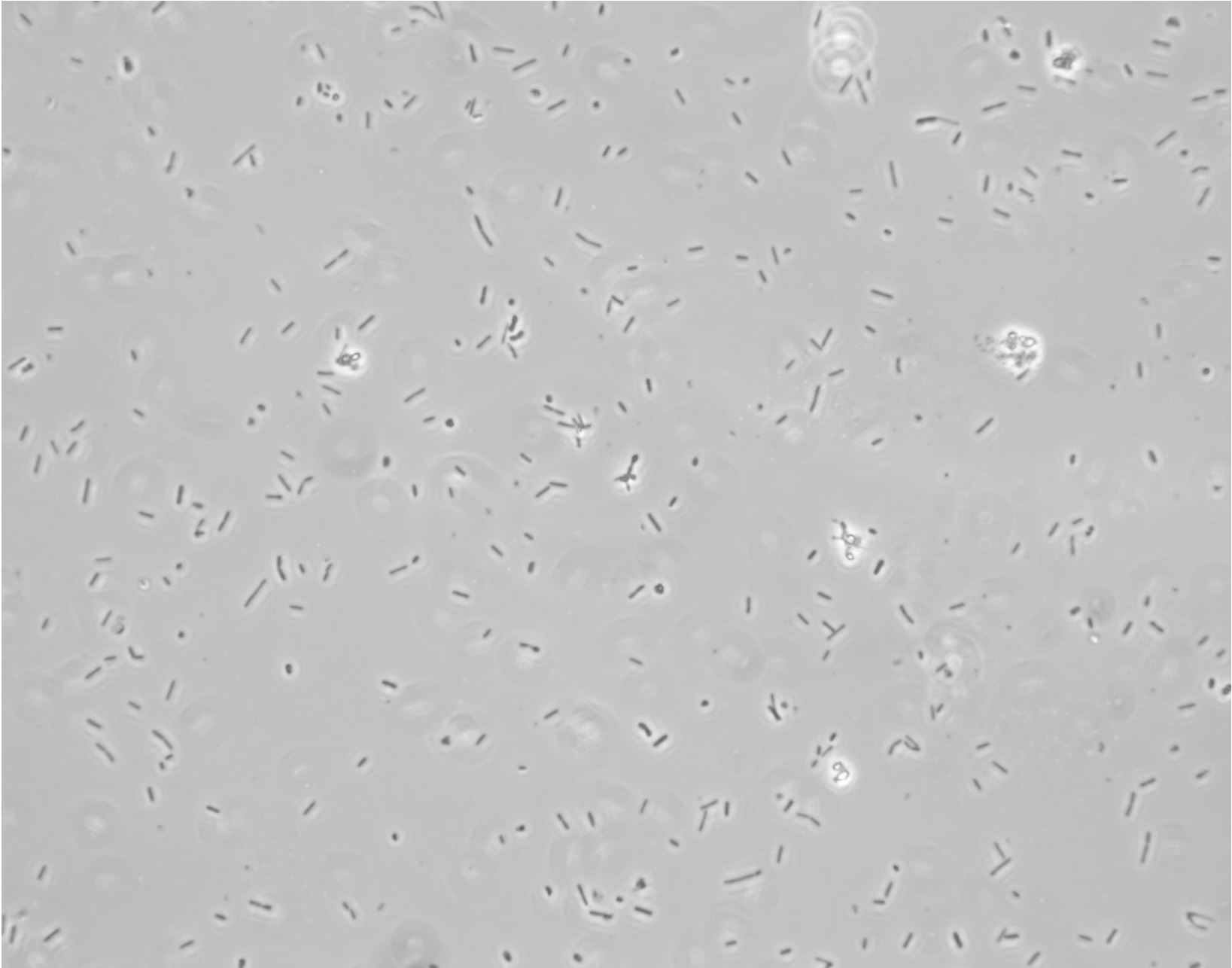

**Delta-Sph4 - HVO\_B0173 spät (OD<sub>600</sub> = 1.6) -1**

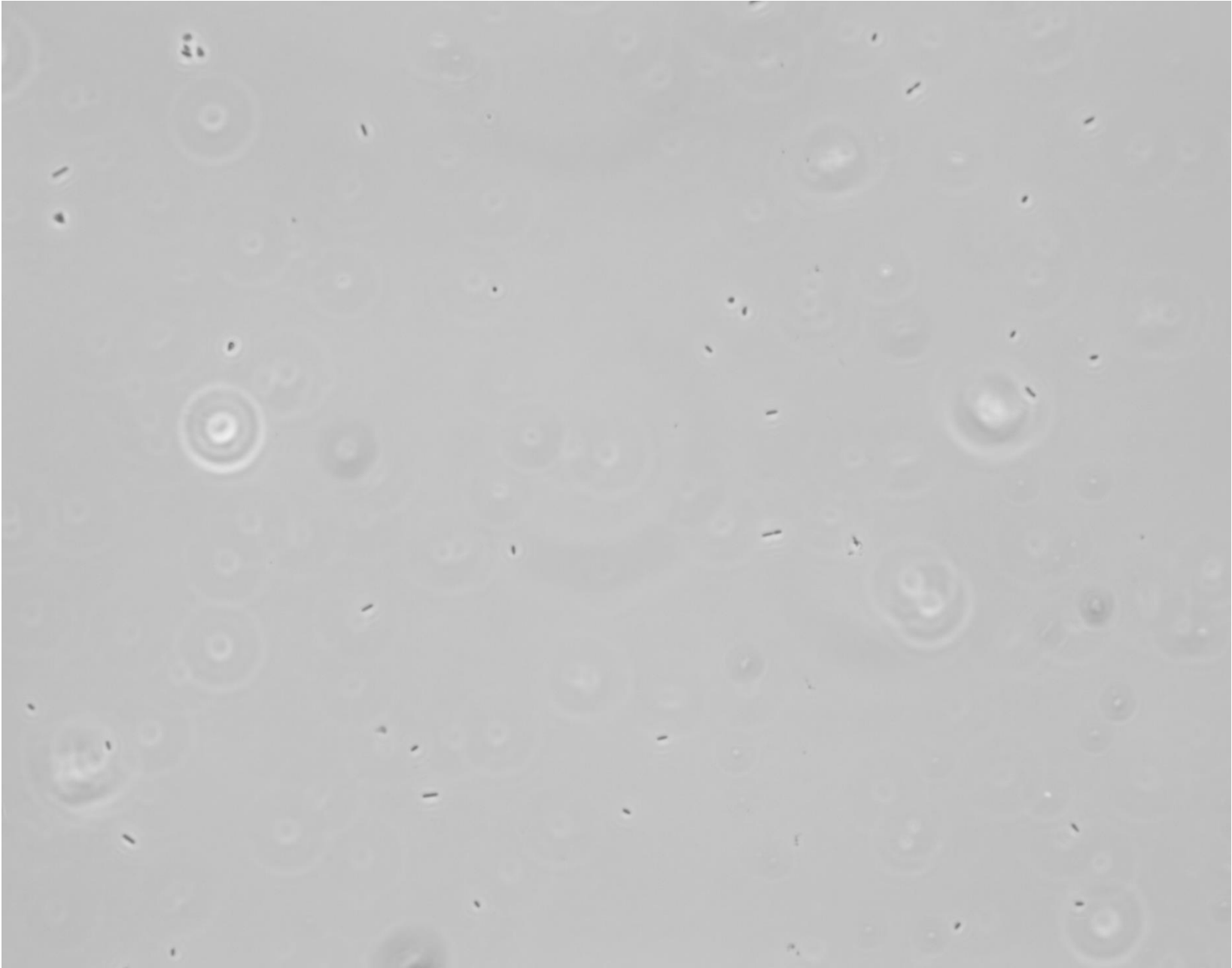

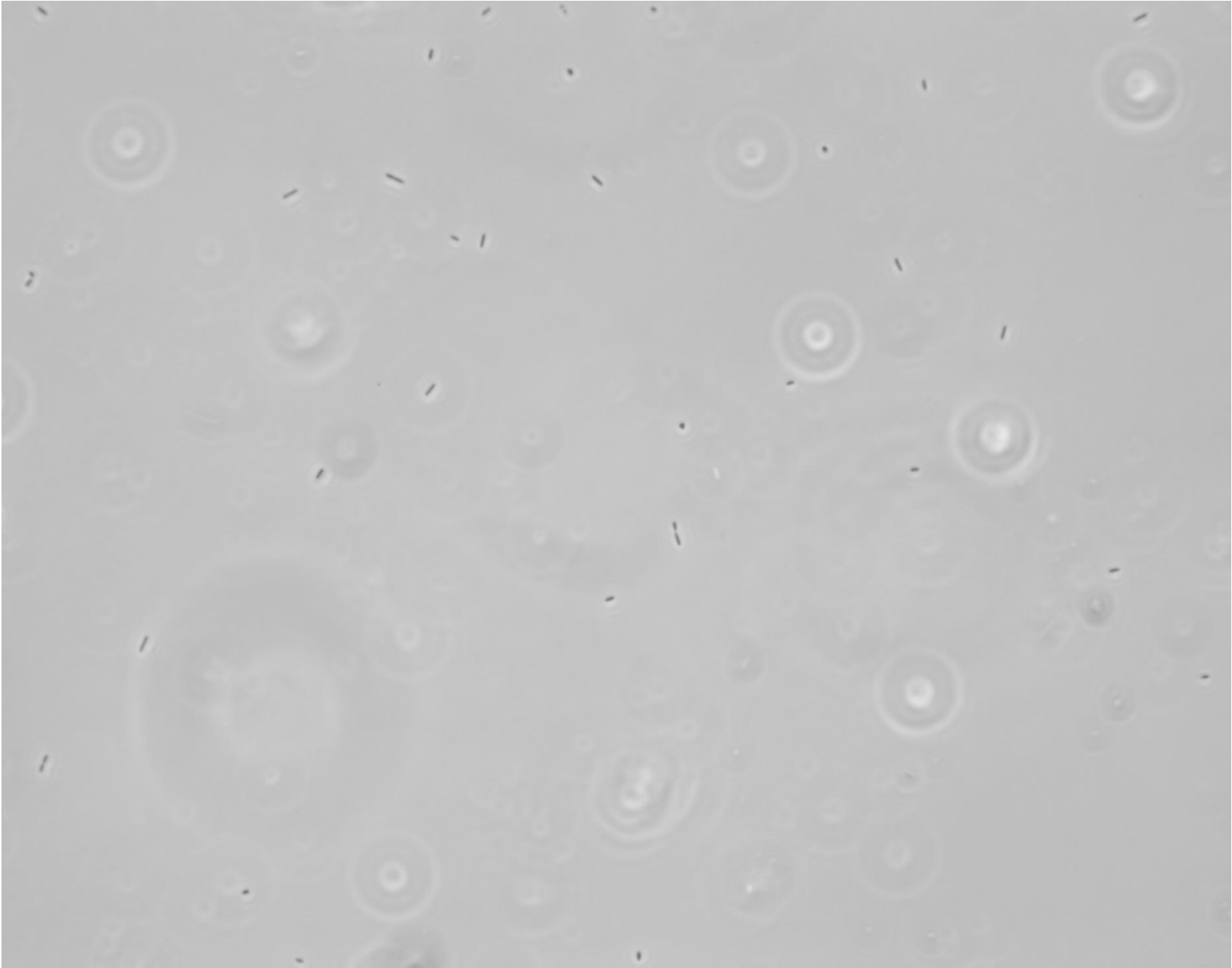

Supplement: Supplementary file 1 [file genes-15-00861-s001.zip › Supplementary Material/Supplementary_Figure_S11.pdf]

Suppl. Fig. S1

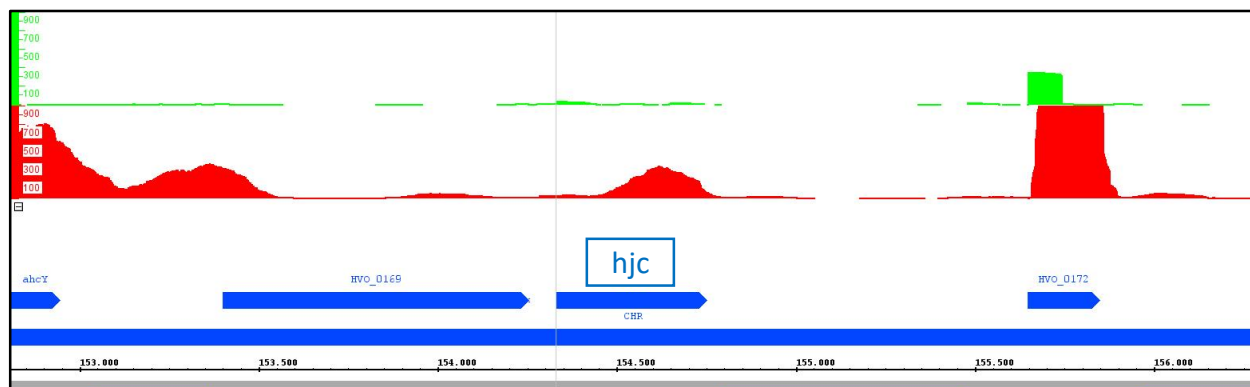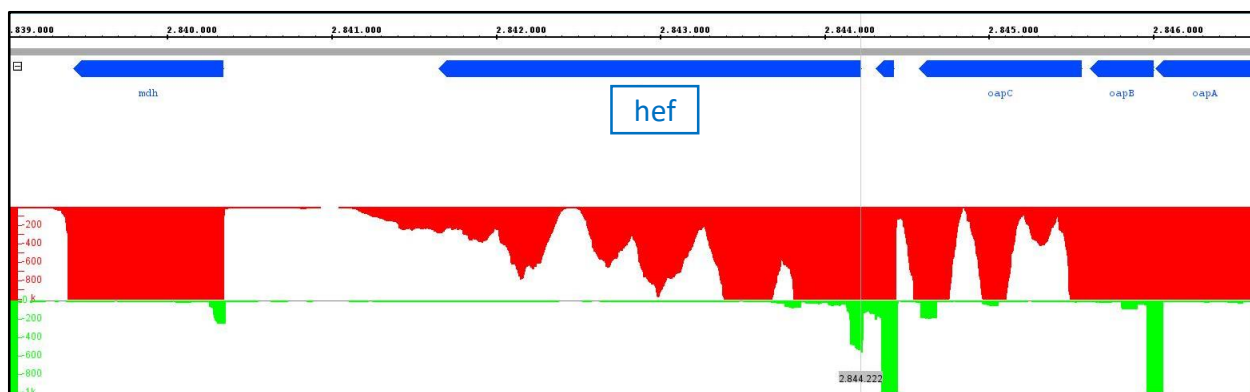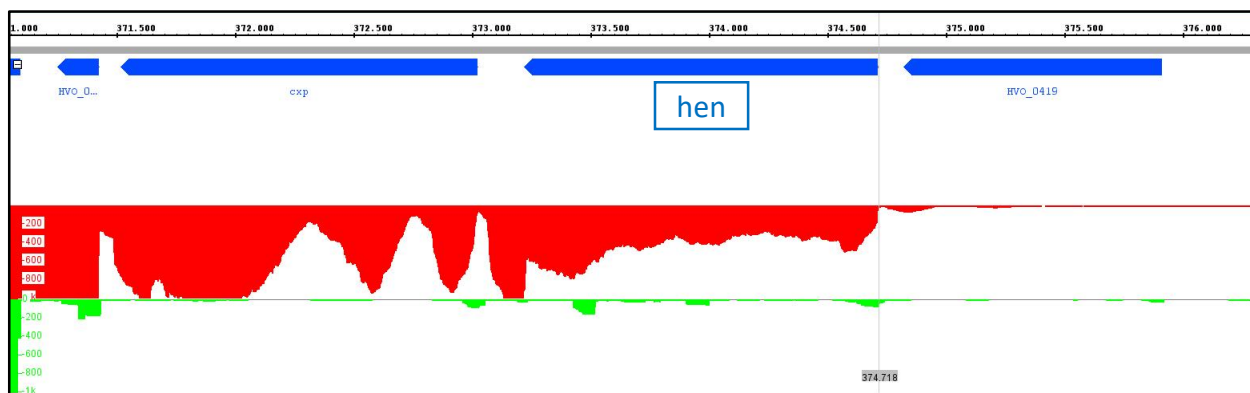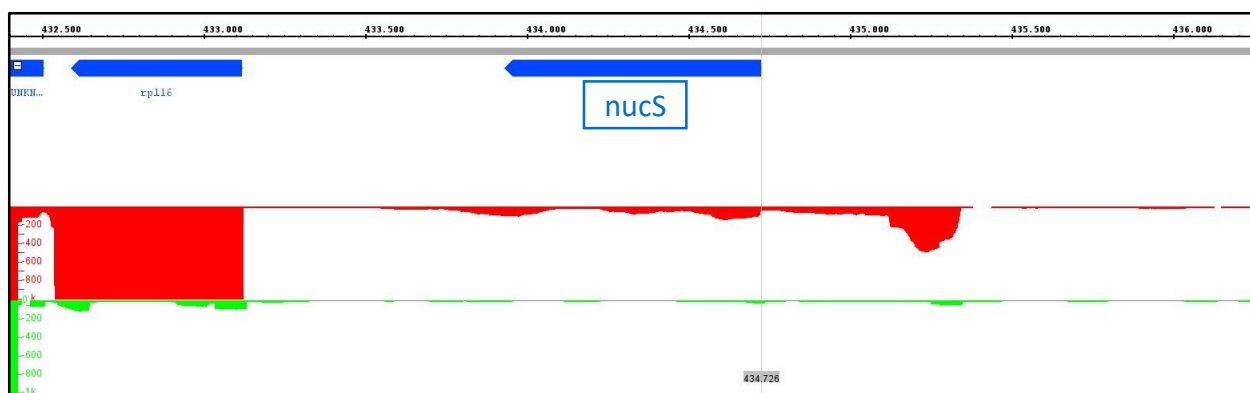

Supplement: Supplementary file 1 [file genes-15-00861-s001.zip › Supplementary Material/Supplementary_Figure_S1.pdf]

Suppl. Fig. S2A

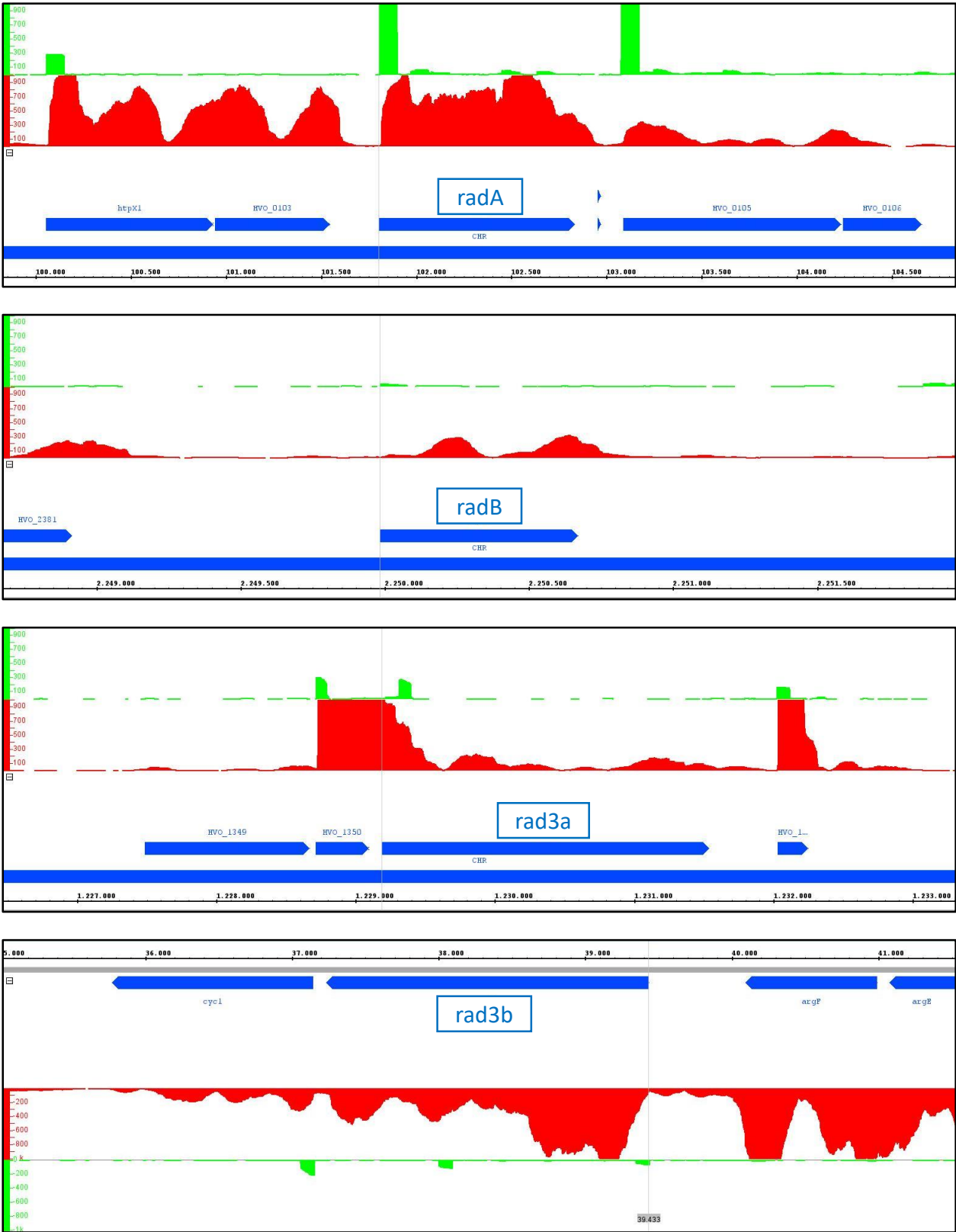

Supplement: Supplementary file 1 [file genes-15-00861-s001.zip › Supplementary Material/Supplementary_Figure_S2A.pdf]

Suppl. Fig. S2B

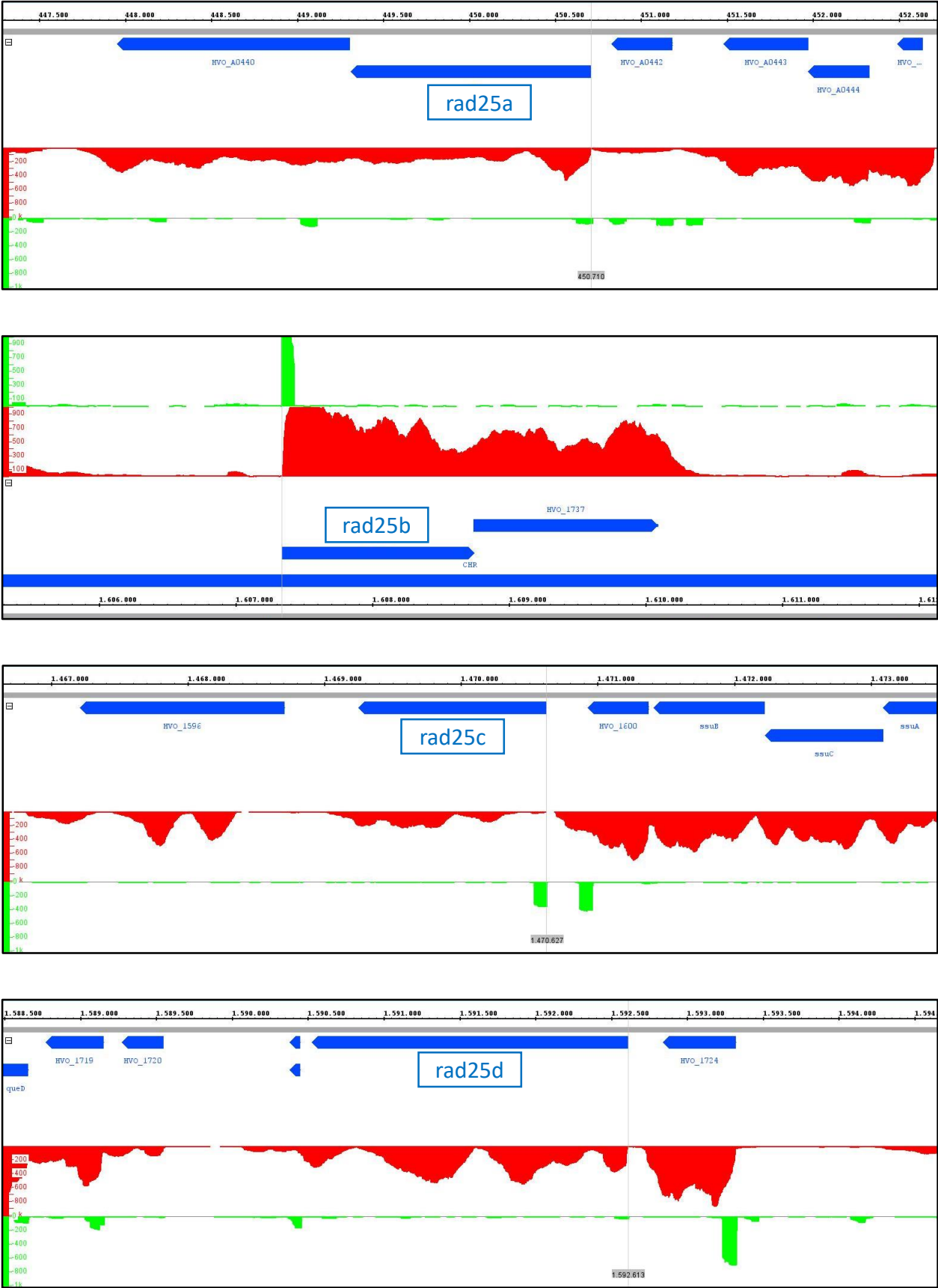

Supplement: Supplementary file 1 [file genes-15-00861-s001.zip › Supplementary Material/Supplementary_Figure_S2B.pdf]

Suppl. Fig. S3

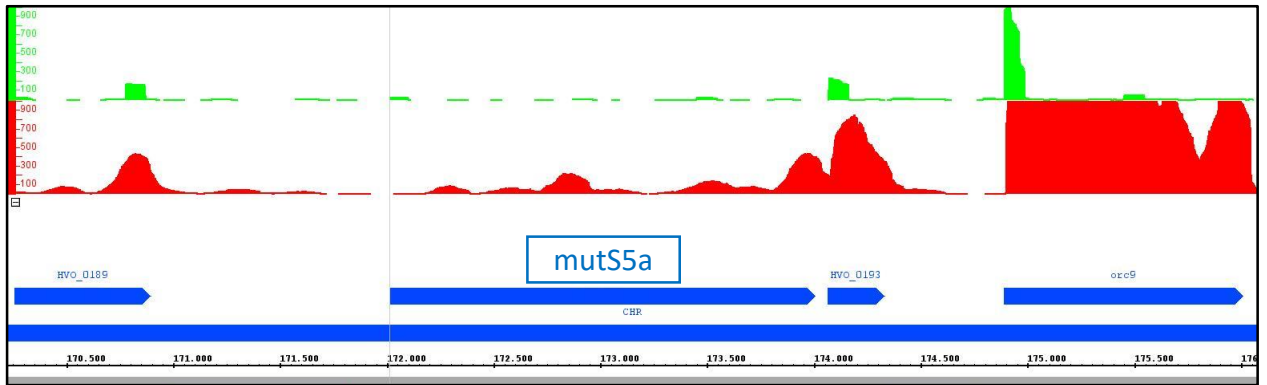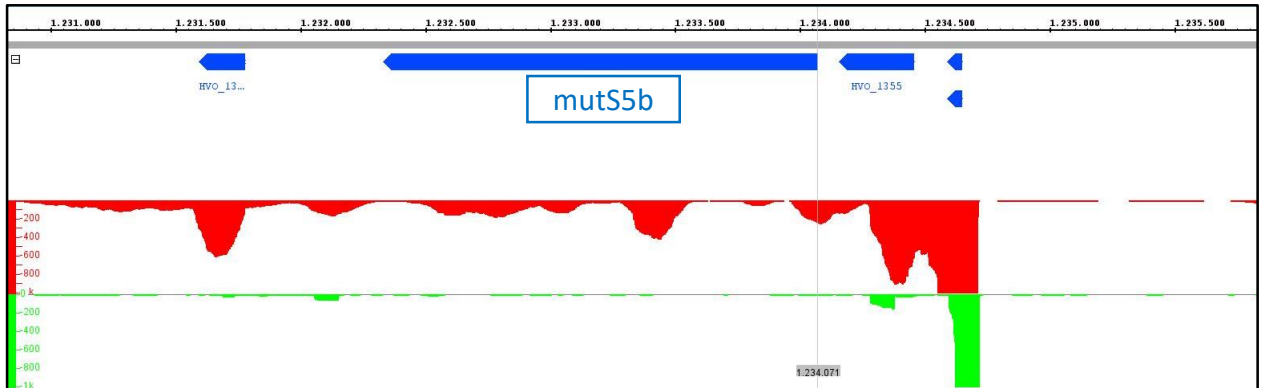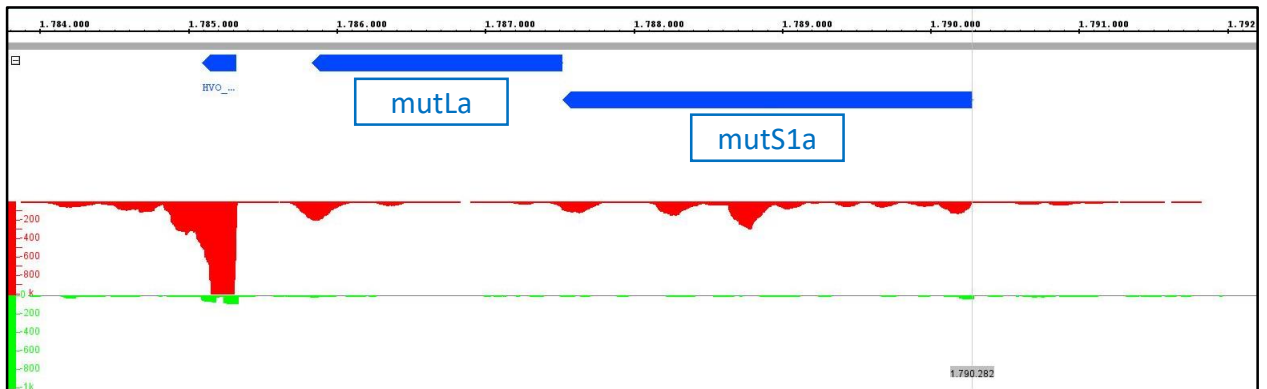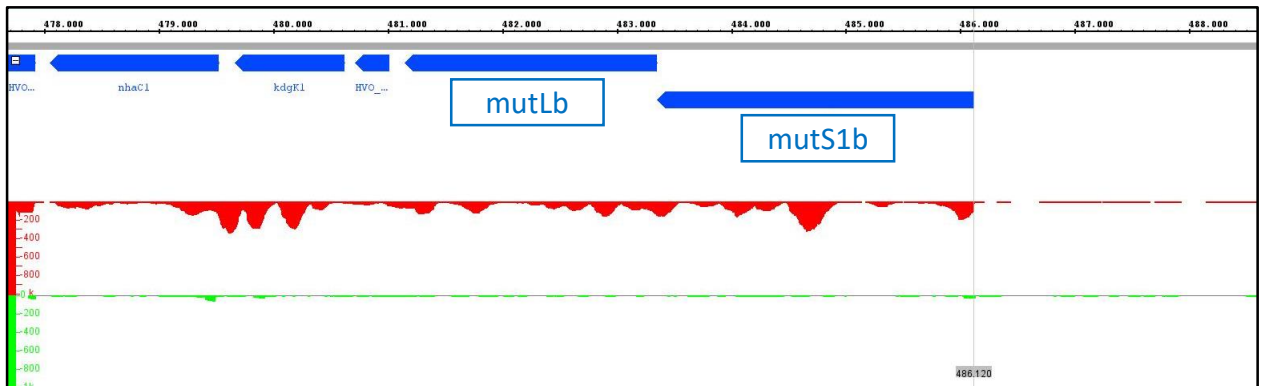

Supplement: Supplementary file 1 [file genes-15-00861-s001.zip › Supplementary Material/Supplementary_Figure_S3.pdf]

Suppl. Fig. S4

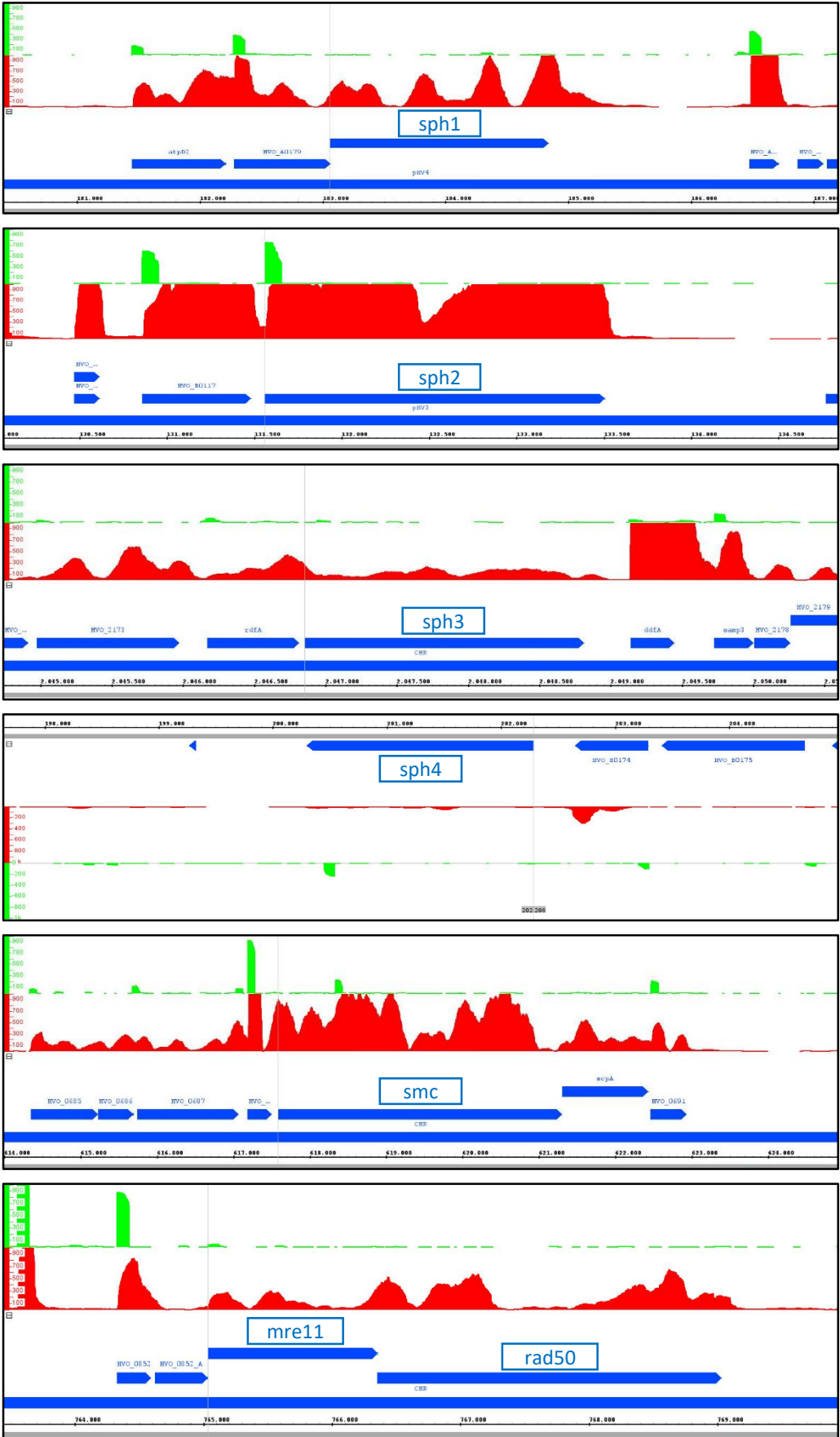

Supplement: Supplementary file 1 [file genes-15-00861-s001.zip › Supplementary Material/Supplementary_Figure_S4.pdf]
